# DUSP10 upregulation is a poor prognosticator and promotes cell proliferation and migration in glioma

**DOI:** 10.3389/fonc.2022.1050756

**Published:** 2023-01-11

**Authors:** Fang Zhou, Lingfeng Zeng, Xi Chen, Fan Zhou, Zhen Zhang, Yixiao Yuan, Heping Wang, Huayi Yao, Jintao Tian, Xujie Liu, Jinxi Zhao, Xiaobin Huang, Jun Pu, William C. Cho, Jianxiong Cao, Xiulin Jiang

**Affiliations:** ^1^ Hunan University of Chinese Medicine, Changsha, China; ^2^ Department of Oncology, the Affiliated Hospital of Hunan Academy of Traditional Chinese Medicine, Changsha, China; ^3^ Carol & Richard Yu Peritoneal Dialysis Research Centre, Department of Medicine and Therapeutics, Prince of Wales Hospital, Shatin, Hong Kong, Hong Kong SAR, China; ^4^ Li Ka Shing Institute of Health Sciences (LiHS), Faculty of Medicine, The Chinese University of Hong Kong, Shatin, Hong Kong, Hong Kong SAR, China; ^5^ Department of Neurosurgery, The Second Affiliated Hospital of Kunming Medical University, Kunming, China; ^6^ Department of Hematology, The Second Affiliated Hospital of Kunming Medical University, Kunming, Yunnan, China; ^7^ Key Laboratory of Molecular Oncology and Epigenetics, The First Affiliated Hospital of Chongqing Medical University, Chongqing, China; ^8^ Department of Clinical Oncology, Queen Elizabeth Hospital, Hong Kong, Hong Kong SAR, China; ^9^ Department of Oncology, The First Hospital of Hunan University of Chinese Medicine, Changsha, China; ^10^ Kunming College of Life Science, University of Chinese Academy of Sciences, Beijing, China

**Keywords:** glioma, DUSP10, prognosticator, cell proliferation, cell migration

## Abstract

Dual-specificity phosphatase 10 (DUSP10) correlates with inflammation, cytokine secretion, cell proliferation, survival, and apoptosis. However, its role in glioma is unclear. Herein, we sought to examine the expression and the underlying carcinogenic mechanisms of DUSP10 action in glioma. DUSP10 expression in glioma was significantly higher than that in normal brain tissues. High DUSP10 expression indicated adverse clinical outcomes in glioma patients. Increased DUSP10 expression correlated significantly with clinical features in glioma. Univariate Cox analysis showed that high DUSP10 expression was a potential independent marker of poor prognosis in glioma. Furthermore, DUSP10 expression in glioma correlated negatively with its DNA methylation levels. DNA methylation level of DUSP10 also correlated negatively with poor prognosis in glioma. More importantly, DUSP10 expression correlated positively with the infiltration of B cells, CD4+ T cells, CD8+ T cells, neutrophils, macrophages, and dendritic cells in glioma. Gene set enrichment analysis (GSEA) and Kyoto Encyclopedia of Genes and Genomes (KEGG) enrichment analysis confirmed that DUSP10 participated in signaling pathways involved in focal adhesion, TNF cascade, Th17 cell differentiation, and NF-kappa B cascade. Finally, we uncovered that DUSP10 was dramatically upregulated in glioblastoma (GBM) cells and that the knockdown of DUSP10 inhibited glioma cell proliferation and migration. Our findings suggested that DUSP10 may serve as a potential prognostic biomarker in glioma.

## Introduction

Glioma is the most common primary tumor in the brain, accounting for 81% of intracranial malignancies (1, 2). In 2016, the World Health Organization classified glioma into four histopathological grades based on its degree of progression. Grades I and II comprise low-grade glioma (LGG), while grades III and IV suggest high-grade glioma. Oligodendrogliomas and astrocytomas are of grade II type. Anaplastic oligodendrogliomas, anaplastic astrocytomas, anaplastic oligoastrocytomas, and anaplastic ependymomas are grade III type, while glioblastoma (GBM) is of grade IV, the most malignant type (3). Glioma is extremely harmful to the human body; the median survival time of a newly diagnosed glioma patient is merely 12–18 months (4, 5). Although several treatment options for gliomas, including surgery, chemotherapy, radiotherapy, and immunotherapy, are currently employed, the survival rate of these patients remains very low. This is attributed to the heterogeneity of tumors and the complexity owing to epigenetics, making it difficult to determine the therapeutic targets. Further, the physiological blood–brain barrier limits the effects of drugs. Moreover, the infiltrative nature of the tumor cells renders surgical treatment largely ineffective. Therefore, an in-depth understanding of the biological behaviors of tumor occurrence and progression is expected to facilitate more innovative and effective methods for clinical diagnosis and treatment of patients with glioma.

RNA methylation is an important epigenetic modification in eukaryotic cells and plays a pivotal role in systems development and disease progression (6, 7). RNA methylation modification is a dynamic biological process, which mainly involves three different components, including the “writers”, “erasers,” and “reader” (8–10). Recently, diverse studies have revealed the special correlation between tumor microenvironment (TME) infiltrating immune cells and m6A modification, which cannot be explained *via* the RNA degradation mechanism. Emerging work has shown that immune checkpoint blockade (ICB) therapy is effective against advanced human cancer; however, only a small subset of cancer patients could benefit from anti-programmed cell death-1/programmed death ligand 1 (anti-PD-1/PD-L1) immunotherapy (11). Therefore, there is an urgent need to identify factors that can modulate ICB responses. A previous study identified that YTDHF1 appears to be significantly correlated with dendritic cells (DCs) in the tumor microenvironment. Genetic ablation of YTHDF1 in mice leads to reduced tumor growth associated with increased tumor infiltration by cytotoxic T cells while simultaneously reducing infiltration of myeloid-derived suppressor cells (MDSCs) (12).

Dual-specificity phosphatase 10 (DUSP10) can dephosphorylate p38 and the c-jun N-terminal kinase (JNK) (13). Accumulating evidence shows that DUSP10 plays an important role in cancer progression. For example, DUSP10 was highly expressed in colorectal cancer and promotes colorectal cancer cell proliferation by regulating the YAP signaling pathway (14). In pancreatic cancer, inhibition of the function of miR-92a repressed the proliferation of pancreatic cancer cells. Further, miR-92a enhanced the activation of the JNK signaling pathway by directly targeting the JNK signaling inhibitor DUSP10 (15). Moreover, DUSP10 plays a critical role in constraining innate IL-33-mediated cytokine production (16). These findings indicate that DUSP10 might play a critical role in the occurrence of cancer and is a potential therapeutic target. However, information available on the expression, regulation, clinical significance, and biological function of DUSP10 in glioma is scarce.

This study, for the first time, analyzed the role of DUSP10 across diverse cancer types. The expression of DUSP10 and its correlation with clinical characteristics, prognosis, immune cell infiltration, and immunomodulator-related molecules expression, along with its potential functions and mechanisms underlying glioma, were assessed using public databases. Quantitative real-time polymerase chain reaction (RT-qPCR), cholecystokinin octapeptide (CCK8), and transwell assays were performed to measure the effect of the knockdown of DUSP10 in glioma cell growth and migration. Our findings suggested that DUSP10 was a prognostic marker of glioma and might be a potential target for the treatment of these patients.

## Materials and methods

### Expressional analysis

We downloaded the clinical information and RNA expression data of glioma patients (LGG+GBM) from The Cancer Genome Atlas (TCGA; https://www.cancer.gov/tcga/) or TCGA-LGG/GBM cohort. Using these data, we analyzed the expression and clinical features of DUSP10 in glioma.

### Prognostic analysis

The prognoses based on the expression of DUSP10 in glioma patients were analyzed using TCGA-LGG/GBM cohort. Validation of the prognostic value of DUSP10 expression in glioma was performed using the Gene Expression Omnibus (GEO) datasets (https://www.ncbi.nlm.nih.gov/geo). Specifically, the RNA expression data and clinical information of patients were downloaded from the GEO datasets, GSE4271 and GSE4412. The receiver operating characteristic (ROC) curve was plotted using the R package “time ROC”, and the prognostic efficiency was evaluated according to the area under the curve (AUC).

### DNA methylation analysis

In this study, Gene Set Cancer Analysis (GSCA; http://bioinfo.life.hust.edu.cn/GSCA/#/) database was used to analyze the relationship between DUSP10 DNA methylation levels and its expression and prognostic significance in glioma (17).

### GSEA and Kyoto Encyclopedia of Genes and Genomes analysis

The LinkedOmics database (http://www.linkedomics.org/admin.php) was employed to obtain the genes correlated positively or negatively with DUSP10 expression in glioma patients (18). Gene Ontology (GO) and Kyoto Encyclopedia of Genes and Genomes (KEGG) enrichment analyses, along with gene set enrichment analysis (GSEA), were performed to functionally annotate the relevant genes and assess the enriched signaling pathways.

### Immune cell infiltration analysis

The Tumor Immune Estimation Resource (TIMER) (https://cistrome.shinyapps.io/timer/) database was used to determine the correlation between the abundances of six immune cell infiltrates and DUSP10 expression in glioma samples (19). Pearson’s correlation analysis was used to determine the relationship between DUSP10 expression and immunomodulator-related genes in glioma.

### Cell culture and qPCR analysis

Human glioma cell lines (U251, A172, and T98G) and normal human astrocyte (NHA) cells were obtained from the Chinese Academy of Sciences Cell Bank (CASCB, China). NHA cells were cultured in the NHA culture medium (Astrocyte Medium), while U251, A172, and T98G lines were cultured in Dulbecco’s Modified Eagle Medium supplemented with 10% fetal bovine serum. NHA, U251, A172, and T98G cells were grown in a sterile cell incubator at 37°C with 5% CO_2_. Total RNA was isolated from NHA cells and glioma (U251, A172, and T98G) cells using the TRIzol reagent (Invitrogen, Carlsbad, CA, USA) following the manufacturer’s protocol. The qPCR primer sequences for DUSP10 were as follows: F: ATCGGCTACGTCATCAACGTC and R: TCATCCGAGTGTGCTTCATCA. Relative mRNA levels were calculated using the 2^−ΔΔCt^ method. Each experiment was conducted using independent triplicates.

### Transwell assay

The indicated cells were collected and resuspended in a serum-free medium. Next, 2 × 10^4^ cells were seeded into a pre-packed chamber (Corning, New York, NY, USA), and the chamber was inserted into a well containing 20% serum in a 24-well plate. After 24 h of incubation, the cells were fixed with 4% paraformaldehyde and stained with 0.1% crystal violet.

### Cellular apoptosis assay

For the cellular apoptosis assay, indicated cells were harvested and then fixed with 70% ethanol at 4°C overnight. The fixed cells were washed with cold phosphate-buffered saline (PBS), stained with Annexin-V (BD Pharmingen, San Diego, CA, USA) and propidium iodide (PI), and then examined by a flow cytometer.

### Statistical analysis

Comparisons between groups were performed using the Wilcoxon rank-sum or Kruskal–Wallis test. The survival curves were generated using the Kaplan–Meier method and compared for statistically significant differences using the log-rank test. The association between clinical factors and overall survival was assessed using the Cox regression model in the survival analysis. The Kaplan–Meier survival curves were plotted and compared between subgroups using the log-rank test with R packages “survival” and “survminer”. The R package “meta” was used for meta-analysis. The ROC curve, sensitivity, specificity, and AUC were obtained using the R package “pROC”. p-Value < 0.05 was considered significant for all statistical analyses.

## Results

### DUSP10 is highly expressed in glioma

**Figure 1:**
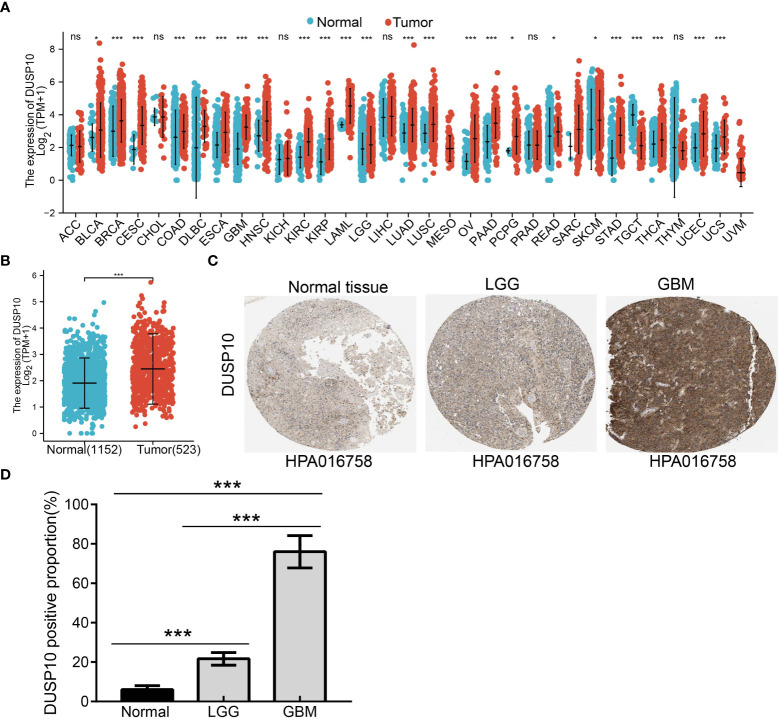
The expression level of DUSP10 in different tumors. **(A)** The expression level of DUSP10 gene in different cancers or specific cancer subtypes was analyzed through TCGA/GTEx database. **(B)** DUSP10 expression in glioma and paired normal tissue in TCGA/GTEx database. **(C, D)** Differential expression of DUSP10 in glioma and normal tissues in the Human Protein Atlas database. NS: p >0.05. * p < 0.05, ** p < 0.01, *** p < 0.001. TCGA, The Cancer Genome Atlas; GTEx, Genotype-Tissue Expression ns:p>0.05.

**Figure 2:**
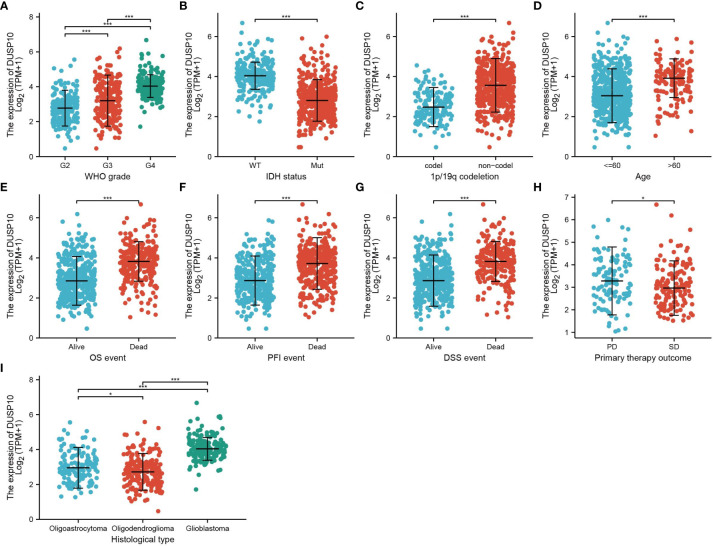
Analysis of the relationship between DUSP10 expression and the clinical features of patients included in TCGA glioma dataset **(A–I)**. Analysis of the relationship between DUSP10 expression and the clinical features of patients included in TCGA glioma dataset, including WHO grade, IDH, 1p/19q codel, age, clinical outcomes events, and histological type. * p < 0.05, *** p < 0.001. IDH, isocitrate dehydrogenase; WT, wild type; Mut, mutant; OS, overall survival; PFS, progression-free survival; DSS, disease-free survival; PD, progressive disease; SD, stable disease.

With the use of TCGA and Genotype-Tissue Expression (GTEx) databases, the expression of DUSP10 was found to be enhanced in various cancers, especially glioma (**Figures 1A** **, B**). Moreover, immunohistochemical images of the normal brain tissue, low-grade glioma, and high-grade glioma acquired from the Human Protein Atlas (HPA) database confirmed the elevated protein levels of DUSP10, which increased with tumor grade (**Figures 1C** **, D**). In TCGA-LGG/GBM dataset, a significant increase in DUSP10 expression was observed in WHO grades IV and III relative to grade II (**Figure 2A**). In patients with isocitrate dehydrogenase (IDH) mutation and 1p/19q co-deletion, DUSP10 expression decreased significantly (**Figures 2B** **, C**). Patients showing higher DUSP10 expression in glioma were >60 years (**Figure 2D**). High expression of DUSP10 correlated significantly with the clinical outcomes and histological type in glioma (**Figures 2E–I** and **Table 1**).

**Table 1:** Clinical characteristics of glioma patients in TCGA dataset according to DUSP10 expression.

Characteristic	Low expression of DUSP10	High expression of DUSP10	p
n	348	348	
WHO grade, n (%)			<0.001
G2	176 (27.7%)	48 (7.6%)	
G3	123 (19.4%)	120 (18.9%)	
G4	10 (1.6%)	158 (24.9%)	
IDH status, n (%)			<0.001
WT	23 (3.4%)	223 (32.5%)	
Mut	323 (47.1%)	117 (17.1%)	
1p/19q codeletion, n (%)			<0.001
Codel	146 (21.2%)	25 (3.6%)	
Non-codel	202 (29.3%)	316 (45.9%)	
Primary therapy outcome, n (%)			0.001
PD	55 (11.9%)	57 (12.3%)	
SD	94 (20.3%)	53 (11.5%)	
PR	48 (10.4%)	16 (3.5%)	
CR	97 (21%)	42 (9.1%)	
Age, n (%)			<0.001
≤60	318 (45.7%)	235 (33.8%)	
>60	30 (4.3%)	113 (16.2%)	
Histological type, n (%)			<0.001
Astrocytoma	99 (14.2%)	96 (13.8%)	
Glioblastoma	10 (1.4%)	158 (22.7%)	
Oligoastrocytoma	87 (12.5%)	47 (6.8%)	
Oligodendroglioma	152 (21.8%)	47 (6.8%)	
OS event, n (%)			<0.001
Alive	289 (41.5%)	135 (19.4%)	
Dead	59 (8.5%)	213 (30.6%)	
DSS event, n (%)			<0.001
Alive	289 (42.8%)	142 (21%)	
Dead	53 (7.9%)	191 (28.3%)	
PFS event, n (%)			<0.001
Alive	234 (33.6%)	116 (16.7%)	
Dead	114 (16.4%)	232 (33.3%)	
Age, median	39 (31,49)	54 (39,63)	<0.001

IDH, isocitrate dehydrogenase; WT, wild type; Mut, mutant; PD, progressive disease; SD, stable disease; PR, partial remission; CR, complete remission; OS, overall survival; PFS, progression-free survival; DSS, disease-free survival; TCGA, The Cancer Genome Atlas.

### Prognostic and multivariate analyses

**Figure 3:**
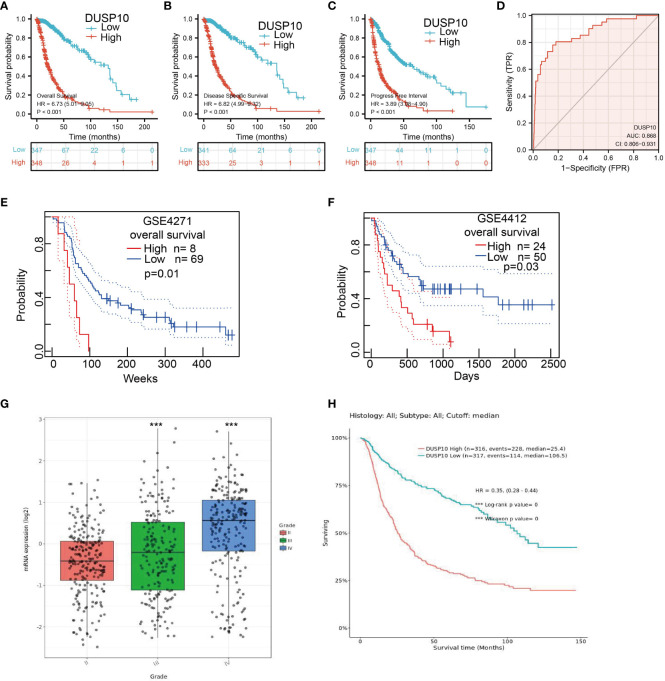
Kaplan–Meier survival analysis, and ROC curve of DUSP10 in glioma. **(A–C)** Kaplan–Meier survival analysis correlated high expression of DUSP10 with the poor prognosis of OS, DFS, and PFS for glioma patients using TCGA databases. **(D)** ROC curve analysis to evaluate the prognostic value of DUSP10 expression in glioma examined by TCGA database. **(E, F)** Kaplan–Meier survival analysis correlated high expression of DUSP10 with the poor prognosis of OS for glioma patients using the GEO database. **(G, H)** CGGA database was used to validate the grade and prognosis of DUSP10 in glioma. *** p < 0.001. ROC, receiver operating characteristic; OS, overall survival; DFS, disease-specific survival; PFS, progression-free survival; GEO, Gene Expression Omnibus; CGGA, Chinese Glioma Genome Atlas.

**Figure 4:**
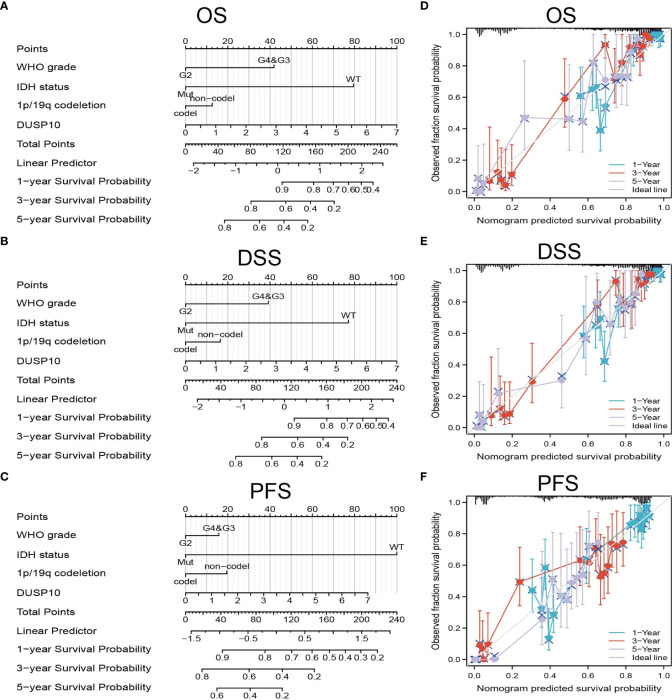
Construction and performance validation of the DUSP10-based nomogram for glioma patients. **(A–C)** The nomogram was constructed based on four factors for predicting 1-, 3-, or 5-year overall survival, disease-specific survival, and progression-free survival of patients with glioma in TCGA database. **(D–F)** The calibration plots of internal validation in TCGA dataset showed good consistency in predicting 1-, 3-, or 5-year overall survival, disease-specific survival, and progression-free survival of patients with glioma in TCGA database. TCGA, The Cancer Genome Atlas.

We conducted a survival analysis by retrieving the clinical information of glioma patients in TCGA. The results showed that glioma patients with the higher DUSP10 expression had poor overall survival (OS), disease-specific survival (DSS), and progression-free survival (PFS) (**Figures 3A** **–C**). We analyzed the diagnostic utility of DUSP10 expression using ROC curves (**Figure 3D**). The GEO glioma datasets also indicated that high expression of DUSP10 was closely associated with adverse clinical outcomes in these patients (**Figures 3E** **, F**). We used the Chinese Glioma Genome Atlas (CGGA) database to validate the grade and prognosis of DUSP10 in glioma, and we found that DUSP10 expression was correlated with tumor grade and adverse clinical outcomes (**Figures 3G** **–H**). We performed univariate and multivariate Cox analyses and found that DUSP10 expression was an independent prognostic indicator in the univariate Cox analysis model (**Table 2**). We also established a nomogram to integrate DUSP10 expression as a glioma biomarker. The higher total points on the nomogram for OS, PFS, and DSS, indicated a worse prognosis (**Figures 4A–C**, respectively). Therefore, this model showed good correspondence between the predicted and observed values (**Figures 4D** **–F**).

**Table 2:** Univariate and multivariate analyses of DUSP10 and clinical features in TCGA datasets.

Characteristics	Total (N)	Univariate analysis		Multivariate analysis	
		Hazard ratio (95% CI)	p-Value	Hazard ratio (95% CI)	p-Value
WHO grade	466				
G2	223				
G3	243	3.059 (2.046–4.573)	<0.001	0.672 (0.220–2.053)	0.486
IDH status	685				
WT	246				
Mut	439	0.117 (0.090–0.152)	<0.001	0.078 (0.014–0.421)	0.003
1p/19q codeletion	688				
Non-codel	518				
Codel	170	0.226 (0.147–0.347)	<0.001	4.730 (0.442–50.642)	0.199
Primary therapy outcome	259				
PD	112				
SD	147	0.437 (0.292–0.654)	<0.001	0.198 (0.066–0.600)	0.004
Age	695				
≤60	552				
>60	143	4.668 (3.598–6.056)	<0.001	1.140 (0.342–3.799)	0.831
Histological type	302				
Oligoastrocytoma	134				
Glioblastoma	168	9.903 (6.394–15.339)	<0.001		
DUSP10	695	2.220 (1.968–2.503)	<0.001	1.503 (0.818–2.762)	0.018

### Correlation of DUSP10 expression and its DNA methylation status

**Figure 5:**
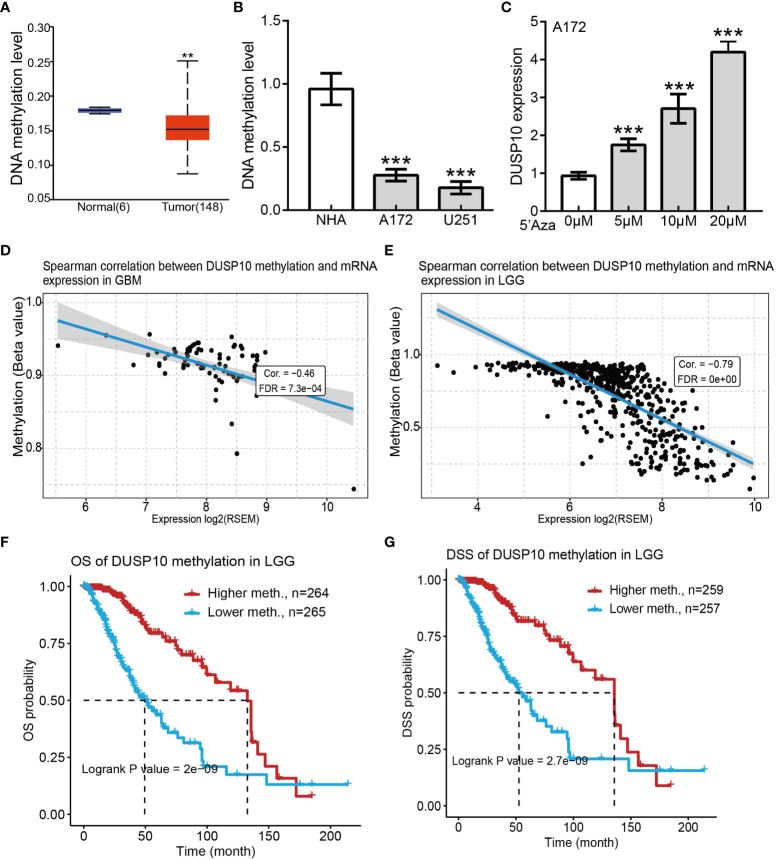
Correlations between DUSP10 mRNA expression and DNA Methylation based on GSCA. **(A)** Mean methylation levels of the DUSP10 promoter in glioma versus normal tissues in TCGA cohort. **(B)** Mean methylation levels of the DUSP10 promoter in glioma cells versus normal human astrocytes examined by Methy-PCR. **(C)** DUSP10 expression in A172 cells was significantly up-regulated in a dose-dependent manner after 5-azacytidine treatment. **(D–E)** DUSP10 expression negatively correlates with mean DUSP10 promoter methylation levels in TCGA cohort. **(F-G)** Correlation between DNA methylation and glioma patients clinical outcomes. ** p < 0.01, *** p < 0.001. GSCA, Gene Set Cancer Analysis; TCGA, The Cancer Genome Atlas.

DNA methylation plays a crucial role in regulating gene expression. We examined the methylation status of the DUSP10 promotor region in glioma using the sequencing data of TCGA-glioma cohort. The mean level of DNA methylation was significantly lower in glioma tissues than in normal tissues (**Figure 5A**). Methylation-specific PCR (MSP) assay confirmed that the mean level of DNA methylation was significantly higher in normal human astrocyte cells than in human glioma cell lines (**Figure 5B**). To further evaluate whether hypomethylation enhanced DUSP10 expression, we performed *in vitro* experiments by adding 5-azacytidine to A172 cells. 5-Azacytidine significantly upregulated DUSP10 expression in a dose-dependent manner (**Figure 5C**). Additionally, the regression analysis revealed a significant negative correlation between DUSP10 expression and its DNA methylation status (**Figures 5D** **, E**). The DUSP10 DNA methylation-low group was associated with poorer OS and DSS as compared to the high group (**Figures 5F** **, G**). Taken together, these results suggested that DNA methylation is an important mechanism modulating DUSP10 expression in glioma.

### GSEA and KEGG analysis for DUSP10

**Figure 6:**
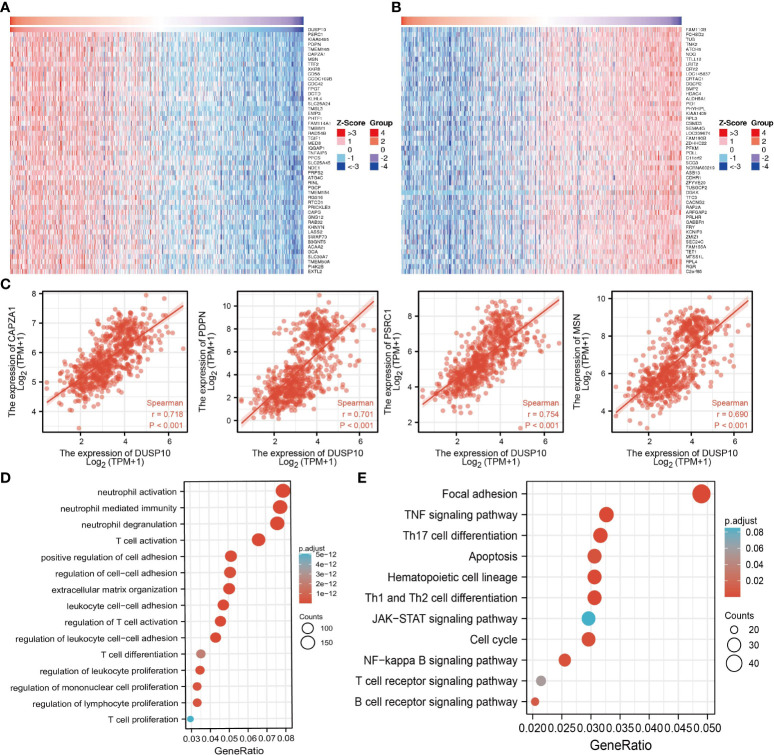
Functional analysis of DEGs between the high and low DUSP10 expression groups in TCGA dataset. **(A, B)** Heatmaps of the differentially expressed genes between the high and low DUSP10 expression groups. **(C)** The top eight coexpressed genes of DUSP10 were selected, and correlation analysis was performed in the online database. **(D, E)** GO and KEGG enrichment analyses of the high and low DUSP10 expression groups in TCGA dataset. DEGs, differentially expressed genes; TCGA, The Cancer Genome Atlas; GO, Gene Ontology; KEGG, Kyoto Encyclopedia of Genes and Genomes.

To assess the biological functions in the progression of glioma, we conducted GO and KEGG enrichment analyses. Using LinkedOmics, we obtained the top 100 genes that were positively or negatively associated with DUSP10 expression in glioma based on TCGA cohort (**Figures 6A** **–C**). GO analysis revealed that high expression of DUSP10 was mainly related to neutrophil activation, T-cell activation, positive regulation of cell adhesion, regulation of T-cell activation, T-cell differentiation, and T-cell proliferation (**Figure 6D**). KEGG results showed that DUSP10 was mainly involved in focal adhesion, TNF signaling pathway, Th17 cell differentiation, apoptosis, Th1, and Th2 cell differentiation, JAK-STAT signaling pathway, cell cycle, NF-kappa B signaling pathway, T-cell receptor signaling pathway, and B-cell receptor signaling pathway (**Figure 6E**).

**Figure 7:**
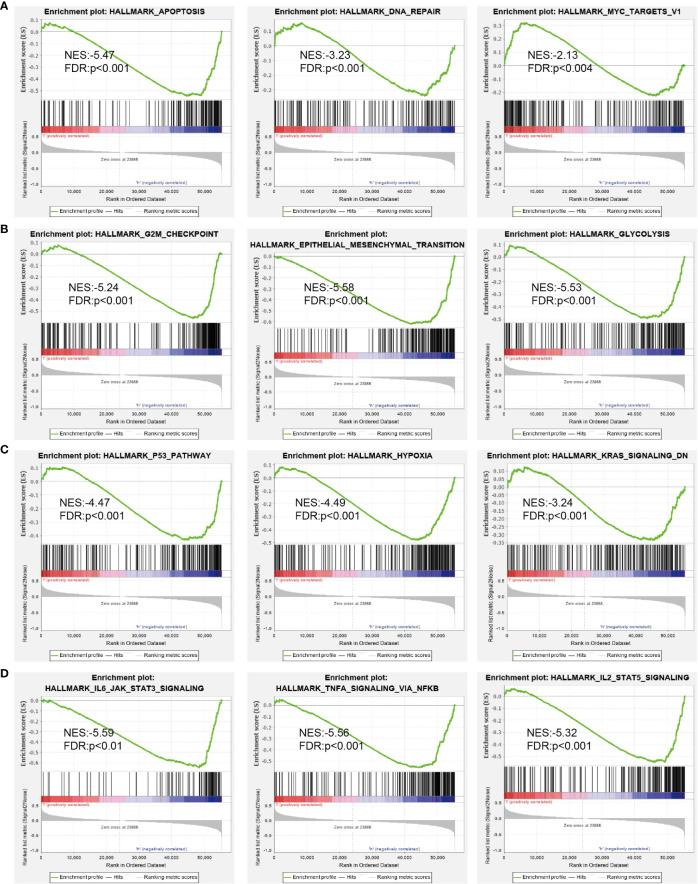
GSEA of DUSP10 based on expression in TCGA-glioma dataset **(A–D)**. DUSP10 was divided into high- and low-expression groups, and GSEA was performed. NES, normalized enrichment score; NOM p-value, nominal p-value; FDR q-val, false discovery rate; GSEA, gene set enrichment analysis; TCGA, The Cancer Genome Atlas.

Next, we screened the potential signaling pathways related to DUSP10 expression in glioma by GSEA. Based on the enrichment scores, apoptosis, DNA repair, glycolysis, G2/M checkpoints, p53 pathway, hypoxia, Kras pathway, and IL-2 STAT5 signaling pathway were found to be significantly positively correlated with the high DUSP10 expression phenotype (**Figures 7A** **–D**).

### Correlation of DUSP10 expression and immune cell infiltration

**Figure 8:**
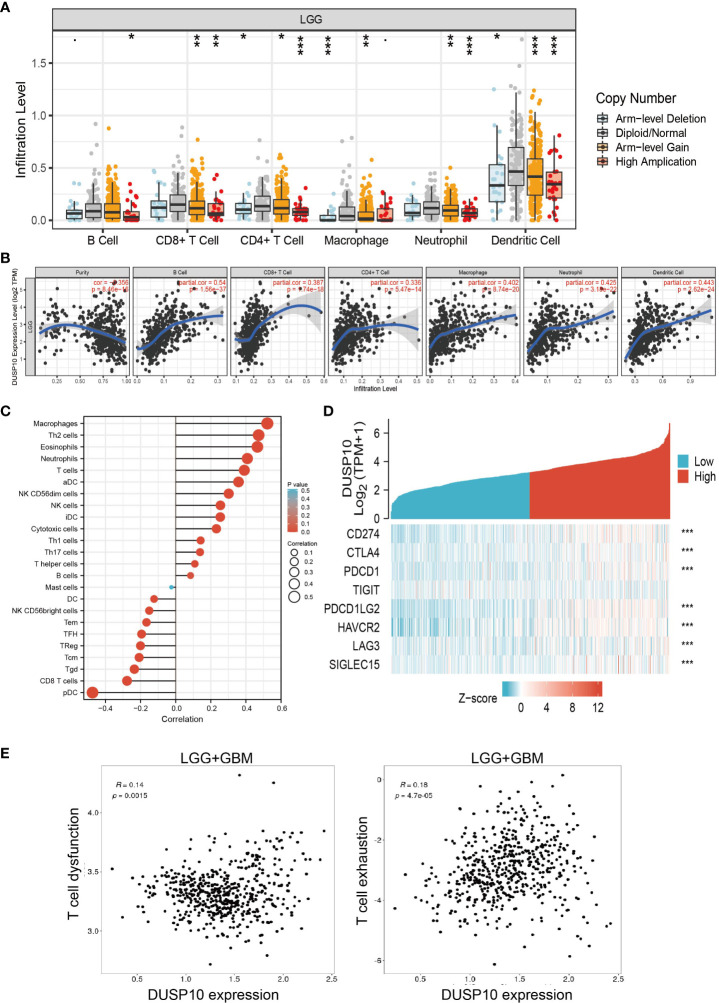
Relationships with DUSP10 in the tumor immune microenvironment from TCGA database. **(A, B)** DUSP10 is significantly associated with tumor purity and is positively correlated with the infiltration of different immune cells using the TIMER database. **(C)** Correlation analysis of DUSP10 gene expression and infiltration of various types of immune cells. **(D)** Correlation analysis of DUSP10 gene expression and the immune checkpoint biomarkers. **(E)** Correlation between immune cell and glioma patients’ clinical outcomes. * p < 0.05, ** p < 0.01, *** p < 0.001. TCGA, The Cancer Genome Atlas.

The prognosis of glioma patients is related to the infiltration and activation of immune cells (20). Given that tumor-infiltrating immune cells are crucial for cancer treatment progression, we analyzed the correlation between DNA copy alterations of DUSP10 and the infiltration of three immune cell types using the TIMER algorithm and found a significant correlation with immune infiltration levels of B cells, CD4+ T cells, CD8+ T cells, neutrophils, macrophages, and dendritic cells in glioma (**Figure 8A**). Furthermore, DUSP10 expression correlated positively with B cells, CD4+ T cells, CD8+ T cells, neutrophils, macrophages, and dendritic cells (**Figures 8B** **, C**). We also found a strong positive correlation between immunomodulator-related genes and DUSP10 expression in glioma (**Figure 8D**). Finally, using the Tumor Immunotherapy Gene Expression Resource (TIGER) database, we found that DUSP10 expression was positively correlated with T-cell dysfunction and T-cell exhaustion in glioma (**Figure 8E**).

### Knockdown of DUSP10 inhibited GBM cell growth and migration

**Figure 9:**
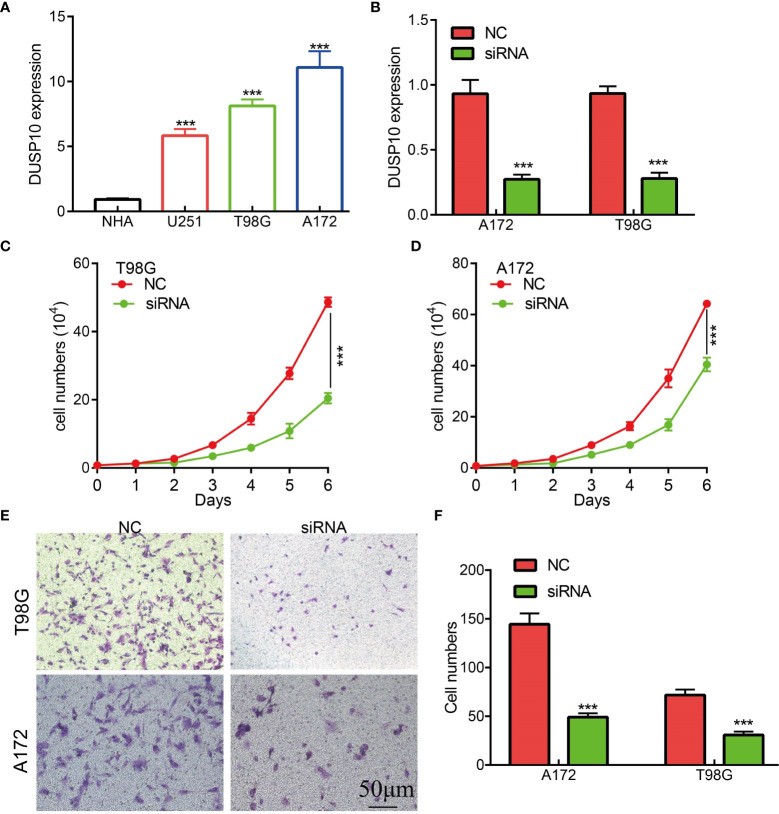
Knockdown of DUSP10 inhibited glioma cell growth and migration. **(A)** qRT-PCR assay indicated that the expression of DUSP10 was upregulated in glioma cells compared with that in normal human astrocyte (NHA) cells. **(B)** The DUSP10 knockdown efficiency of different siRNAs in A172 and T98G cells. **(C–F)** Knockdown of DUSP10 significantly decreased the growth and migration capabilities of glioma cells. NC, negative control; siRNA, DUSP10 siRNA. *** p < 0.001. .

To date, no study has examined whether DUSP10 expression correlates with glioma progression. The results of qRT-PCR results showed that DUSP10 expression was significantly higher in GBM cell lines (U251, T98G, and A172) compared to the NHA cells (**Figure 9A**). Given that DUSP10 is upregulated in glioma, we knocked down DUSP10 expression using short interfering RNA (siRNA), and its efficiency was verified by a real-time RT-PCR assay (**Figure 9B**). Knocking down DUSP10 significantly decreased the growth and migration of GBM cells (**Figures 9C** **–F**). Collectively, these results demonstrated that the expression of DUSP10 correlated positively with glioma cell proliferation and migration.

## Discussion

Due to the high heterogeneity of gliomas, there is high variability across individuals (21). Therefore, the treatment of glioma needs comprehensive consideration based on individual prognostic factors, clinical symptoms, side effects, and tumor progression (22). The genetic examination can be used to guide radiotherapy and chemotherapy. For an instance, people with mutations in the isocitrate dehydrogenase 1 (IDH1) and 2 (IDH2) genes have a more favorable prognosis and clinical response to radiotherapy and chemotherapy (23). Moreover, the methylated methylation of *O* ^6^-methylguanine-DNA methyltransferase (MGMT) status has a predictive value for the benefit of chemotherapy, and the 1p19q co-deletion status is considered unsuited for radiotherapy (24, 25); however, biomarkers to guide adjuvant immunotherapy are lacking. Thus, we aimed to assess the underlying mechanism of DUSP10 action in the progression of gliomas and its potential immune activation and sensitivity to immunotherapeutic responses in these patients.

RNA expression of DUSP10 is downregulated in the liver and hematopoietic systems (26). Microarray expression data show that myeloid and T cells have the highest expression of DUSP10. Shi et al. described that DUSP10 is a negative regulator of muscle stem cell function in mice, decreasing cell proliferation and myogenesis by selective p38 and JNK dephosphorylation (14). DUSP10 is an inducible phosphatase during immune responses (26). Furthermore, DUSP10 is highly expressed in colorectal cancer (CRC) cell lines and promotes CRC cell proliferation *via* the regulator of yes-associated protein 1 (YAP1) activity (14). In line with the above findings, it appears that high expression of DUSP10 may be involved in the progression of cancer. However, the relationship between DUSP10 expression and the clinicopathological characteristics of glioma patients, as well as the prognostic significance of DUSP10 expression for glioma, has not been well studied.

In the present study, DUSP10 was found to be highly expressed across multiple cancer types, especially glioma. We observed that patients with high levels of DUSP10 showed shorter OS, PFS, and DSS relative to those with low DUSP10 expression. To further assess the correlation between DUSP10 expression and glioma, the data from GEO were divided into high and low DUSP10 subgroups according to the median value of DUSP10 expression. The results showed that high DUSP10 expression was more likely to be associated with a poor prognosis in these patients. ROC analysis demonstrated that DUSP10 expression was a reliable marker for predicting the clinical outcomes in glioma. Furthermore, univariate and multivariate Cox analyses identified DUSP10 expression as an independent prognostic risk factor for glioma. Therefore, we speculated that DUSP10 expression could serve as a predictor for the clinical prognosis of glioma patients.

Previous studies have confirmed that epidermal growth factors and hypoxia may induce high DUSP10 expression in diverse cells (27, 28). MiR-21, miR-30b, and miR-155 bind to the 3′-untranslated region of DUSP10 and inhibit the expression of DUSP10 in diverse cancer cells (29). As an important epigenetic modification, DNA methylation plays a crucial role in regulating gene expression (30). In this study, we focused on the genetic or epigenetic alterations regulating DUSP10 expression. By analyzing TCGA data, hypomethylation of the DUSP10 promoter was found to be associated with its elevated expression in glioma tissues. In the *in vitro* study, we demonstrated that DUSP10 upregulation was mediated by DNA demethylation that drove glioma malignancy.

We conducted KEGG functional enrichment analyses to identify the underlying regulatory mechanisms, and DUSP10 was found to be primarily associated with focal adhesion, TNF signaling pathway, Th17 cell differentiation, apoptosis, Th1 and Th2 cell differentiation, JAK-STAT signaling pathway, cell cycle, NF-kappa B signaling pathway, T-cell receptor signaling pathway, and B-cell receptor signaling pathway. These results suggested that DUSP10 expression promoted glioma development through numerous immune-related pathways or biological processes in addition to affecting the cell proliferation of these cells. These pathways are known to be closely related to glioma malignancy. For example, the JAK-STAT signaling pathway is an important signaling pathway for immune monitoring and homeostasis. It plays an important role in the immune system of organisms. If not controlled properly, the immune system can also act against healthy cells (31). Because glioma patients often show lower immunity, we have reason to believe that it is closely related to the complement system. Cytokines are key factors in the TME, exerting an immunosuppressive function and inflammatory activity along with participating in the progression of GBM (32). Moreover, focal adhesion plays a role in tumor invasion and metastases, and the interaction between the extracellular matrix (ECM) and the glioma microenvironment is an important contributor to its malignant progression (33).

The prognosis of glioma is related to the infiltration and activation of immune cells (34). Immunotherapies can markedly improve patient survival and have shown significant antitumor outcomes in several clinical trials (47). The TME of glioma patients is shaped by the disease and not by the surrounding brain tissue. The innate immune system, instead of CD8+ T cells, might hold greater responsibility for the therapeutic effects of anti-PD-1 antibodies against glioblastoma. In glioblastoma, severe T-cell exhaustion induces upregulation of multiple immune checkpoints, in turn inhibiting immune modulation (35). Furthermore, not all patients with glioma can benefit from monotherapy immune checkpoint inhibition (36). Therefore, new predictive biomarkers to improve precision immunotherapy for patients with glioma are necessitated. The following bioinformatics analysis showed a close relationship between DUSP10 expression and immune progression, which indicated the role of DUSP10 in the glioma immune microenvironment. In this study, we observed that DUSP10 expression in glioma correlated positively with the abundance of B cells, CD4+ T cells, CD8+ T cells, neutrophils, macrophages, and dendritic cells. We also observed a strong positive correlation between immunomodulator-related genes and DUSP10 expression in glioma. Finally, we showed that DUSP10 expression may affect the clinical outcomes of glioma patients by mediating immune cell infiltration. The above findings suggested a critical role of DUSP10 in the remodeling of the immune component of TME in glioma.

ICB uses immune checkpoint inhibitors to block inhibitory signaling pathways and directly stimulates the activation of cytotoxic T lymphocytes to achieve antitumor effects (37, 38) by promoting the killing ability of T cells against cancer cells. Although the immune system can recognize malignant tumor cells, due to the upregulation of suppressive immune checkpoints in the tumor microenvironment, the inactivation of antitumor T cells leads to ineffective immune responses to cancer. In recent years, immune checkpoint inhibitors have shown significant survival benefits in distinct tumors. Similarly, preclinical studies have shown that immune checkpoint inhibitors have great prospects in the treatment of GBM (39, 40). Therefore, in this study, we also investigated the correlation between DUSP10 expression and several common immune checkpoints. Our results confirmed that DUSP10 expression correlated positively with the expressions of PD-L1 (CD274), cytotoxic T lymphocyte-associated protein 4 (CTLA4), hepatitis A virus cellular receptor 2 (HAVCR2), lymphocyte-activation gene 3 (LAG3), programmed cell death 1 (PDCD1), programmed cell death 1 ligand 2 (PDCD1LG2), T-cell immunoreceptor with Ig and ITIM domains (TIGIT), and sialic acid binding Ig-like lectin 15 (SIGLEC15). Based on the above, we conclude that DUSP10 has great potential in tumor immunotherapy, and targeting DUSP10 and other immune checkpoint molecules is likely to be a novel approach for glioma treatment.

Diverse studies have confirmed the high expression of DUSP10 in human tumor tissues. For example, in human hepatocellular cancer, the expression of DUSP10 is elevated and promotes cancer cell metastasis through enhanced ERK activation (41). DUSP10 is an induced gene in HER2-positive breast tumors (42). In this study, our bioinformatics analysis results showed that DUSP10 expression increased with the increasing degree of malignancy of glioma. The immunohistochemical images obtained from HPA showed that DUSP10 expression was lower in normal brain tissue relative to glioma tissues. Furthermore, the HPA immunohistochemistry images showed that high-grade glioma tissues had higher levels of DUSP10 than low-grade glioma tissues. Moreover, DUSP10 expression in normal human astrocytes was lower than that in human glioma cell lines (U251, A172, and T98G), as evidenced by qRT-PCR analysis. Thus, the above observations confirmed the findings of the bioinformatics analyses. Finally, *in vitro*, functional experiments showed that knocking down DUSP10 inhibited glioma cell proliferation and migration and promotes glioma cell apoptosis glioma cells. Apoptosis has attracted much interest due to its intricate nature and diverse roles in maintaining a healthy and self-sustainable biological entity. DUSP10 inhibited glioma cell proliferation may be by promoting glioma cell apoptosis glioma cells. Thus, DUSP10 might represent a potential target in the treatment of glioma. However, future experimental validation of the biological significance and potential mechanism of DUSP10 action in glioma is needed.

This is the first study to assess the correlation between DUSP10 and glioma. However, some limitations warrant consideration. First, our study was based on expression data extracted from TCGA but may be more convincing if supported by a prospective clinical study. Furthermore, the biological functions of DUSP10 need further *in vivo* experimental validation. In the future, we plan to assess the function of DUSP10 in tumor progression and tumor microenvironment regulation in glioma. We plan to perform more *in vivo* and *in vitro* experiments to assess the function and the potential molecular mechanisms underlying the effects of DUSP10 in tumor progression and tumor microenvironment regulation of glioma.

## Conclusion

In the present study, we found enhanced DUSP10 expression in glioma, which was also associated with a poor prognosis. Furthermore, DUSP10 might be involved in the progression of glioma by regulating the function of immune-infiltrating cells and immune response-related signaling pathways. Herein, we unravel the biological functions of DUSP10 in glioma and offer a potential strategy for the diagnosis and treatment of these patients.

## Data availability statement

The original contributions presented in the study are included in the article/supplementary material. Further inquiries can be directed to the corresponding authors.

## Author contributions

FZ and LZ designed this work and performed related assays. XC, FanZ, ZZ, YY, HW, HY, JT, XL, JZ, XH, and JP analyzed the data. XJ, WC, and JC supervised and wrote the manuscript. All authors have read and approved the final version of the manuscript.
