# Supplementary material for: DUSP10 upregulation is a poor prognosticator and promotes cell proliferation and migration in glioma
Source: Front Oncol. 2023 Jan 11;12:1050756. doi: 10.3389/fonc.2022.1050756 (PMC9874937; doi:10.3389/fonc.2022.1050756)
Supplement: Supplementary file 1 [file DataSheet_1.zip › DUSP10 raw data/Figure 3/3F.pdf]

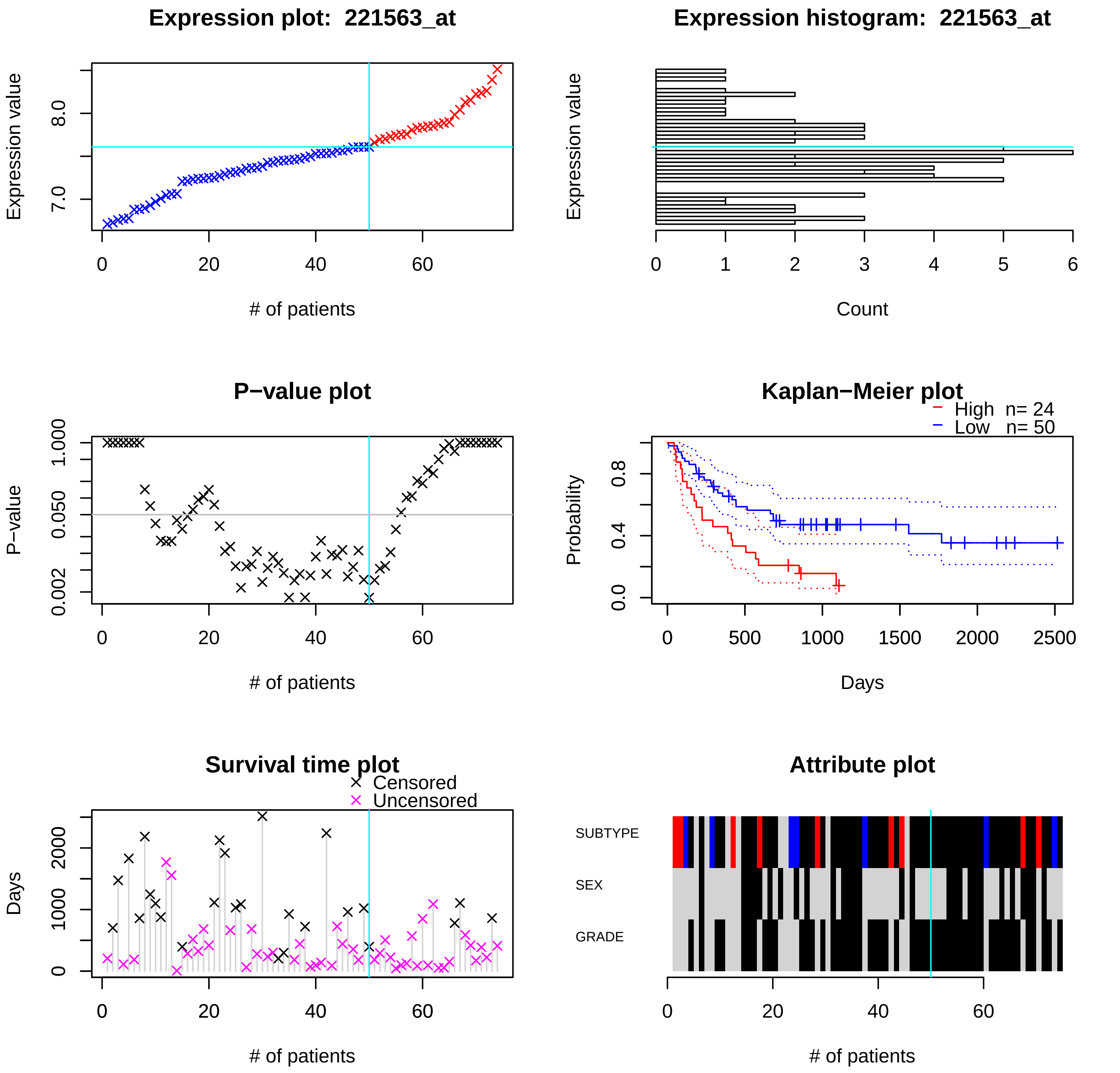
Expression plot: 221563_at
Expression value
# of patients
Expression histogram: 221563_at
Expression value
Count
P−value plot
P−value
# of patients
Kaplan−Meier plot
High n= 24
Low n= 50
Probability
Days
Survival time plot
Censored
Uncensored
Days
# of patients
Attribute plot
SUBTYPE
SEX
GRADE
# of patients
