# Supplementary material for: DUSP10 upregulation is a poor prognosticator and promotes cell proliferation and migration in glioma
Source: Front Oncol. 2023 Jan 11;12:1050756. doi: 10.3389/fonc.2022.1050756 (PMC9874937; doi:10.3389/fonc.2022.1050756)
Supplement: Supplementary file 1 [file DataSheet_1.zip › DUSP10 raw data/Figure 4/4B.pdf]

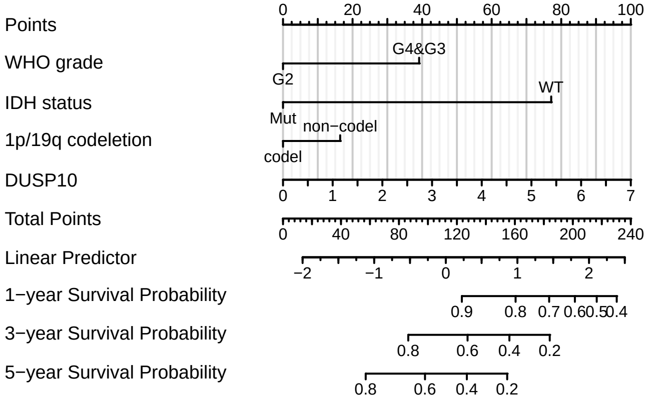
Points
0 20 40 60 80 100
WHO grade
G4&G3
G2
IDH status
WT
Mut
1p/19q codeletion
non−codel
codel
DUSP10
0 1 2 3 4 5 6 7
Total Points
0 40 80 120 160 200 240
Linear Predictor
−2 −1 0 1 2
1−year Survival Probability
0.9 0.8 0.7 0.6 0.5 0.4
3−year Survival Probability
0.8 0.6 0.4 0.2
5−year Survival Probability
0.8 0.6 0.4 0.2
