# Supplementary material for: DUSP10 upregulation is a poor prognosticator and promotes cell proliferation and migration in glioma
Source: Front Oncol. 2023 Jan 11;12:1050756. doi: 10.3389/fonc.2022.1050756 (PMC9874937; doi:10.3389/fonc.2022.1050756)
Supplement: Supplementary file 1 [file DataSheet_1.zip › DUSP10 raw data/Figure 4/4C.pdf]

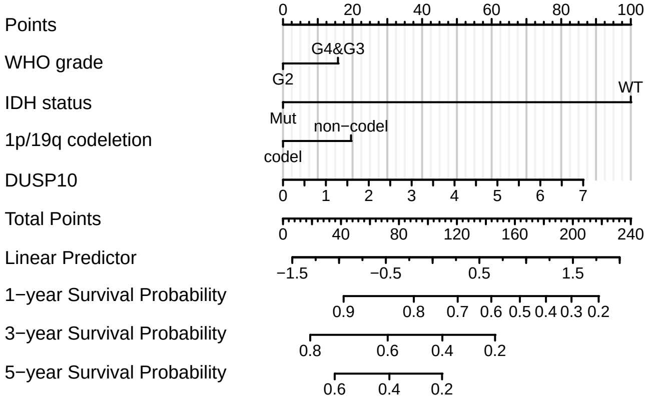

Points
0
20
40
60
80
100
WHO grade
G4&G3
G2
IDH status
WT
Mut
1p/19q codeletion
non−codel
codel
DUSP10
0
1
2
3
4
5
6
7
Total Points
0
40
80
120
160
200
240
Linear Predictor
−1.5
−0.5
0.5
1.5
1−year Survival Probability
0.9
0.8
0.7
0.6
0.5
0.4
0.3
0.2
3−year Survival Probability
0.8
0.6
0.4
0.2
5−year Survival Probability
0.6
0.4
0.2
