# Supplementary material for: DUSP10 upregulation is a poor prognosticator and promotes cell proliferation and migration in glioma
Source: Front Oncol. 2023 Jan 11;12:1050756. doi: 10.3389/fonc.2022.1050756 (PMC9874937; doi:10.3389/fonc.2022.1050756)
Supplement: Supplementary file 1 [file DataSheet_1.zip › DUSP10 raw data/R code.docx]

**Figure 1**

library(tidyverse)

library(ggplot2)

library(reshape2)

library(car)

library(rstatix)

set.seed(100)

data <- data.frame(x = rnorm(100, 2, 1), y = rnorm(100, 1, 1))

data2 <- melt(data)

data3 <- lapply(data, function(x) get_summary_stats(data.frame(x)))

data3

# $x

# # A tibble: 1 x 13

# variable n min max median q1 q3 iqr mad mean sd se ci

# <chr> <dbl> <dbl> <dbl> <dbl> <dbl> <dbl> <dbl> <dbl> <dbl> <dbl> <dbl> <dbl>

# 1 x 100 -0.272 4.58 1.94 1.39 2.66 1.26 0.974 2.00 1.02 0.102 0.203

#

# $y

# # A tibble: 1 x 13

# variable n min max median q1 q3 iqr mad mean sd se ci

# <chr> <dbl> <dbl> <dbl> <dbl> <dbl> <dbl> <dbl> <dbl> <dbl> <dbl> <dbl> <dbl>

# 1 x 100 -1.14 3.17 0.927 0.568 1.45 0.878 0.648 1.01 0.796 0.08 0.158

data3 <- rbind(data3[[1]], data3[[2]])

data3[1] <- c("x", "y")

## Shapiro-Wilk normality test

lapply(data, function(x) shapiro.test(x))

# $x

#

# Shapiro-Wilk normality test

#

# data: x

# W = 0.98836, p-value = 0.535

#

#

# $y

#

# Shapiro-Wilk normality test

#

# data: x

# W = 0.98532, p-value = 0.3348

## Levene's Test

leveneTest(value~variable, data = data2)

# Levene's Test for Homogeneity of Variance (center = median)

# Df F value Pr(>F)

# group 1 4.4476 0.03621 *

# 198

t.test(value~variable, data = data2, var.equal = T)

# Two Sample t-test

#

# data: value by variable

# t = 7.6613, df = 198, p-value = 8.012e-13

# alternative hypothesis: true difference in means is not equal to 0

# 95 percent confidence interval:

# 0.7364913 1.2470521

# sample estimates:

# mean in group x mean in group y

# 2.002913 1.011141

t.test(value~variable, data = data2, var.equal = F)

# Welch Two Sample t-test

#

# data: value by variable

# t = 7.6613, df = 186.92, p-value = 9.657e-13

# alternative hypothesis: true difference in means is not equal to 0

# 95 percent confidence interval:

# 0.7363983 1.2471452

# sample estimates:

# mean in group x mean in group y

# 2.002913 1.011141

wilcox.test(value~variable, data = data2)

# Wilcoxon rank sum test with continuity correction

#

# data: value by variable

# W = 7844, p-value = 3.711e-12

# alternative hypothesis: true location shift is not equal to 0

summary(aov(value~variable, data = data2))

# Df Sum Sq Mean Sq F value Pr(>F)

# variable 1 49.18 49.18 58.7 8.01e-13 ***

# Residuals 198 165.90 0.84

ggplot(data2, aes(x = variable, y = value, color = variable, fill = variable)) +

geom_violin(alpha = 0.2) +

theme_bw()

ggplot(data2, aes(x = variable, y = value, color = variable, fill = variable)) +

geom_violin(alpha = 0.2) +

geom_point(position = position_jitter(0.3)) +

theme_bw()

ggplot(data2, aes(x = variable, y = value, color = variable, fill = variable)) +

geom_boxplot(alpha = 0.2) +

geom_point(position = position_jitter(0.3)) +

theme_bw()

ggplot(data2, aes(x = variable, y = value, color = variable, fill = variable)) +

geom_violin(alpha = 0.1) +

geom_boxplot(alpha = 0.1) +

geom_point(position = position_jitter(0.3)) +

theme_bw()

ggplot() +

geom_violin(data = data2, aes(x = variable, y = value, color = variable, fill = variable), alpha = 0.1) +

geom_errorbar(data = data3, aes(x = variable, ymin=mean-sd, ymax=mean+sd), width = 0.2)

**Figure 2**

library(tidyverse)

library(ggplot2)

library(reshape2)

library(car)

library(rstatix)

set.seed(100)

data <- data.frame(x = rnorm(100, 2, 1), y = rnorm(100, 1, 1))

data2 <- melt(data)

data3 <- lapply(data, function(x) get_summary_stats(data.frame(x)))

data3

# $x

# # A tibble: 1 x 13

# variable n min max median q1 q3 iqr mad mean sd se ci

# <chr> <dbl> <dbl> <dbl> <dbl> <dbl> <dbl> <dbl> <dbl> <dbl> <dbl> <dbl> <dbl>

# 1 x 100 -0.272 4.58 1.94 1.39 2.66 1.26 0.974 2.00 1.02 0.102 0.203

#

# $y

# # A tibble: 1 x 13

# variable n min max median q1 q3 iqr mad mean sd se ci

# <chr> <dbl> <dbl> <dbl> <dbl> <dbl> <dbl> <dbl> <dbl> <dbl> <dbl> <dbl> <dbl>

# 1 x 100 -1.14 3.17 0.927 0.568 1.45 0.878 0.648 1.01 0.796 0.08 0.158

data3 <- rbind(data3[[1]], data3[[2]])

data3[1] <- c("x", "y")

## Shapiro-Wilk normality test

lapply(data, function(x) shapiro.test(x))

# $x

#

# Shapiro-Wilk normality test

#

# data: x

# W = 0.98836, p-value = 0.535

#

#

# $y

#

# Shapiro-Wilk normality test

#

# data: x

# W = 0.98532, p-value = 0.3348

## Levene's Test

leveneTest(value~variable, data = data2)

# Levene's Test for Homogeneity of Variance (center = median)

# Df F value Pr(>F)

# group 1 4.4476 0.03621 *

# 198

t.test(value~variable, data = data2, var.equal = T)

# Two Sample t-test

#

# data: value by variable

# t = 7.6613, df = 198, p-value = 8.012e-13

# alternative hypothesis: true difference in means is not equal to 0

# 95 percent confidence interval:

# 0.7364913 1.2470521

# sample estimates:

# mean in group x mean in group y

# 2.002913 1.011141

t.test(value~variable, data = data2, var.equal = F)

# Welch Two Sample t-test

#

# data: value by variable

# t = 7.6613, df = 186.92, p-value = 9.657e-13

# alternative hypothesis: true difference in means is not equal to 0

# 95 percent confidence interval:

# 0.7363983 1.2471452

# sample estimates:

# mean in group x mean in group y

# 2.002913 1.011141

wilcox.test(value~variable, data = data2)

# Wilcoxon rank sum test with continuity correction

#

# data: value by variable

# W = 7844, p-value = 3.711e-12

# alternative hypothesis: true location shift is not equal to 0

summary(aov(value~variable, data = data2))

# Df Sum Sq Mean Sq F value Pr(>F)

# variable 1 49.18 49.18 58.7 8.01e-13 ***

# Residuals 198 165.90 0.84

# ---

ggplot(data2, aes(x = variable, y = value, color = variable, fill = variable)) +

geom_violin(alpha = 0.2) +

theme_bw()

ggplot(data2, aes(x = variable, y = value, color = variable, fill = variable)) +

geom_violin(alpha = 0.2) +

geom_point(position = position_jitter(0.3)) +

theme_bw()

ggplot(data2, aes(x = variable, y = value, color = variable, fill = variable)) +

geom_boxplot(alpha = 0.2) +

geom_point(position = position_jitter(0.3)) +

theme_bw()

ggplot(data2, aes(x = variable, y = value, color = variable, fill = variable)) +

geom_violin(alpha = 0.1) +

geom_boxplot(alpha = 0.1) +

geom_point(position = position_jitter(0.3)) +

theme_bw()

ggplot() +

geom_violin(data = data2, aes(x = variable, y = value, color = variable, fill = variable), alpha = 0.1) +

geom_errorbar(data = data3, aes(x = variable, ymin=mean-sd, ymax=mean+sd), width = 0.2)

**Figure 3**

if (!requireNamespace("survminer", quietly = TRUE))

install.packages("survminer")

library(survival)

library(survminer)

# data <- lung

# colnames(data)[5] <- "variable"

fit <- survfit(Surv(time, status) ~ variable, data = data)

print(fit)

# Call: survfit(formula = Surv(time, status) ~ variable, data = data)

#

# n events median 0.95LCL 0.95UCL

# variable=1 138 112 270 212 310

# variable=2 90 53 426 348 550

survdiff(Surv(time, status) ~ variable, data = data)

# survdiff(formula = Surv(time, status) ~ variable, data = data)

#

# N Observed Expected (O-E)^2/E (O-E)^2/V

# variable=1 138 112 91.6 4.55 10.3

# variable=2 90 53 73.4 5.68 10.3

#

# Chisq= 10.3 on 1 degrees of freedom, p= 0.001

fit2 <- coxph(Surv(time, status) ~ variable, data = data)

summary(fit2)

# Call:

# coxph(formula = Surv(time, status) ~ variable, data = data)

#

# n= 228, number of events= 165

#

# coef exp(coef) se(coef) z Pr(>|z|)

# variable -0.5310 0.5880 0.1672 -3.176 0.00149 **

#

# exp(coef) exp(-coef) lower .95 upper .95

# variable 0.588 1.701 0.4237 0.816

#

# Concordance= 0.579 (se = 0.021 )

# Likelihood ratio test= 10.63 on 1 df, p=0.001

# Wald test = 10.09 on 1 df, p=0.001

# Score (logrank) test = 10.33 on 1 df, p=0.001

# plot

ggsurvplot(fit = fit, data = data, pval = T)

**Figure 4**

library(tidyverse)

library(survival)

library(readxl)

library(rms)

## read data

data <- read_xlsx("~/file.xlsx")

## tidy data

# data$event <- as.numeric(data$event)

# data$time <- as.numeric(data$time)

### numeric

data$Age <- as.numeric(data$Age)

data$Score <- as.numeric(data$Score)

### factor

data$Sex <- factor(data$Sex, levels = c("Male", "Female"))

data$Grade <- factor(data$Grade, levels = c("0", "1", "2"))

data$Stage <- factor(data$Stage, levels = c("Stage1", "Stage2", "Stage3", "Stage4"))

colnames(data)[4] <- "Weight_loss"

## summary

fit <- survfit(Surv(time, event) ~ Sex, data = data)

fit

# Call: survfit(formula = Surv(time, event) ~ Sex, data = data)

#

# n events median 0.95LCL 0.95UCL

# Sex=Male 138 112 270 212 310

# Sex=Female 90 53 426 348 550

fit <- coxph(Surv(time = time, event = event) ~ Age + Weight_loss + Sex + Grade + Stage + Score, data = data)

summary(fit)

# Call:

# coxph(formula = Surv(time = time, event = event) ~ Age + Weight_loss +

# Sex + Grade + Stage + Score, data = data)

#

# n= 210, number of events= 148

# (18 observations deleted due to missingness)

#

# coef exp(coef) se(coef) z Pr(>|z|)

# Age 0.012963 1.013047 0.009363 1.384 0.16622

# Weight_loss -0.013179 0.986908 0.007049 -1.870 0.06153 .

# SexFemale -0.648482 0.522839 0.181763 -3.568 0.00036 ***

# Grade1 0.247066 1.280264 0.215206 1.148 0.25095

# Grade2 -0.167493 0.845782 0.243006 -0.689 0.49066

# StageStage2 0.447155 1.563856 0.211797 2.111 0.03475 *

# StageStage3 0.791586 2.206894 0.299925 2.639 0.00831 **

# StageStage4 2.083022 8.028691 1.056227 1.972 0.04859 *

# Score -0.013471 0.986619 0.007642 -1.763 0.07795 .

# exp(coef) exp(-coef) lower .95 upper .95

# Age 1.0130 0.9871 0.9946 1.0318

# Weight_loss 0.9869 1.0133 0.9734 1.0006

# SexFemale 0.5228 1.9126 0.3661 0.7466

# Grade1 1.2803 0.7811 0.8397 1.9520

# Grade2 0.8458 1.1823 0.5253 1.3618

# StageStage2 1.5639 0.6394 1.0326 2.3685

# StageStage3 2.2069 0.4531 1.2260 3.9726

# StageStage4 8.0287 0.1246 1.0129 63.6366

# Score 0.9866 1.0136 0.9720 1.0015

#

# Concordance= 0.665 (se = 0.026 )

# Likelihood ratio test= 38.77 on 9 df, p=1e-05

# Wald test = 36.84 on 9 df, p=3e-05

# Score (logrank) test = 39.94 on 9 df, p=8e-06

dat1 = datadist(data)

options(datadist = "dat1")

cph1 <- cph(formula = Surv(time = time, event = event) ~ Age + Weight_loss + Sex + Grade + Stage + Score,

data=data, x=T, y=T, surv = T)

surv <- Survival(cph1)

surv1 <- function(x) surv(365*1,x)

surv2 <- function(x) surv(365*2,x)

fit <- nomogram(cph1, fun = list(surv1, surv2), lp=T,

funlabel = c("1-Year", "2-Year"))

plot(fit)

**Figure 6**

library(clusterProfiler)

library(org.Hs.eg.db) ## org.Mm.eg.db

gene_ids = bitr(geneID = gene_list, fromType = "SYMBOL",

toType = "ENTREZID", OrgDb = "org.Hs.eg.db")

head(gene_ids)

ego <- enrichGO(gene = gene_ids$ENTREZID, OrgDb = "org.Hs.eg.db",

keyType = "ENTREZID", ont = "BP")

# #

# # over-representation test

# #

# #...@organism Homo sapiens

# #...@ontology BP

# #...@keytype ENTREZID

# #...@gene chr [1:209] "9" "125" "332" "366" "597" "652" "730" "771" "776" "820" "890" "891" "983" "991" "1062" "1101" "1111" "1307" "1308" "1311" ...

# #...pvalues adjusted by 'BH' with cutoff <0.05

# #...187 enriched terms found

# 'data.frame': 187 obs. of 9 variables:

# $ ID : chr "GO:0140014" "GO:0000280" "GO:0048285" "GO:0000070" ...

# $ Description: chr "mitotic nuclear division" "nuclear division" "organelle fission" "mitotic sister chromatid segregation" ...

# $ GeneRatio : chr "32/196" "34/196" "35/196" "23/196" ...

# $ BgRatio : chr "264/18670" "407/18670" "449/18670" "151/18670" ...

# $ pvalue : num 7.53e-25 4.63e-21 1.10e-20 2.23e-20 2.61e-19 ...

# $ p.adjust : num 2.33e-21 7.16e-18 1.14e-17 1.72e-17 1.61e-16 ...

# $ qvalue : num 2.01e-21 6.16e-18 9.78e-18 1.48e-17 1.39e-16 ...

# $ geneID : chr "332/652/891/991/1062/1111/3832/3833/4085/4605/4751/6790/7272/9055/9212/9232/9319/9493/9787/10403/10460/11065/22"| __truncated__ "332/652/891/991/1062/1111/3832/3833/4085/4605/4751/6790/7153/7272/9055/9212/9232/9319/9493/9787/10403/10460/110"| __truncated__ "332/652/891/991/1062/1111/3832/3833/4085/4137/4605/4751/6790/7153/7272/9055/9212/9232/9319/9493/9787/10403/1046"| __truncated__ "891/991/1062/3833/4085/4751/7272/9055/9212/9232/9319/9493/9787/10403/10460/23397/24137/51203/55143/64151/81620/81930/146909" ...

# $ Count : int 32 34 35 23 24 28 20 24 17 19 ...

# #...Citation

# Guangchuang Yu, Li-Gen Wang, Yanyan Han and Qing-Yu He.

# clusterProfiler: an R package for comparing biological themes among

# gene clusters. OMICS: A Journal of Integrative Biology

# 2012, 16(5):284-287

#### enrichKEGG(gene = gene_ids$ENTREZID, organism = "hsa", keyType = "ENTREZID")

**Figure 8**

# library(tidyverse)

library(GSVA)

library(clusterProfiler)

library(org.Hs.eg.db)

library(data.table)

library(rtracklayer)

### ssGSEA ######

## table S1 - https://doi.org/10.1016/j.immuni.2013.10.003

## pdf -> table -> read

immunity <- read.csv("~/immunity-cell-gene.csv", header = T)

# CellType AffymetrixID Symbol Gene.Symbol ENTREZ_GENE_ID

# 1 aDC 205569_at LAMP3 LAMP3 27074

# 2 aDC 207533_at CCL1 CCL1 6346

# 3 aDC 210029_at INDO IDO1 3620

# 4 aDC 218400_at OAS3 OAS3 4940

# 5 aDC 219424_at EBI3 EBI3 10148

# 6 B cells 204836_at GLDC GLDC 2731

idx <- !immunity$CellType %in% c("Blood vessels", "Normal mucosa", "SW480 cancer cells", "Lymph vessels")

immunity <- immunity[idx,]

immunity <- immunity %>%

split(., .$CellType) %>%

lapply(., function(x)(x$ENTREZ_GENE_ID))

immunity <- lapply(immunity, unique)

## Ensembl download

anno <- import('~/Homo_sapiens.GRCh38.101.gtf')

anno <- as.data.frame(anno)

anno <- anno[!duplicated(anno$gene_id),]

anno <- merge(anno, gene_symbol, by = "gene_name")

anno <- rbind(anno, data.frame(gene_name = c("KIAA1324", "IGHA1"),

gene_id = c("ENSG00000116299", "ENSG00000211895"),

ENTREZID = c("57535", "3492")))

anno <- anno[!duplicated(anno$gene_id),] ### 37417

anno <- anno[, c("gene_id", "ENTREZID")]

data <- fread("~/tpm.txt") %>%

rename("gene_id" = "V1") %>%

left_join(., anno, by = "gene_id") %>%

filter(!is.na(ENTREZID)) %>%

select(-gene_id) %>%

column_to_rownames("ENTREZID")

data <- log2(data + 1)

immu_cell <- as.data.frame(gsva(as.matrix(data), immunity, method = "ssgsea"))

### bbt plot

data <- read.table("~/file.txt", header = T)

# group aDC B cells CD8 T cells Cytotoxic cells

# 1 1.13092315 0.4709550 0.26202395 0.5944611 0.5130117

# 3 0.55644003 0.1800251 -0.07081909 0.5197230 0.2135559

# 4 0.44696904 0.3350859 0.05579749 0.5908900 0.3135561

# 5 0.05474605 0.1191767 0.02578815 0.5541712 0.2068595

# 7 0.61364297 0.1563856 0.09869185 0.5518254 0.2321028

# 8 0.41079217 0.4588979 0.50105493 0.5996277 0.4866508

data1 <- NULL

for(i in 2:25){

cor <- cor.test(data[,i], data[,1], method = "pearson")

data1 <- rbind(data1,

data.frame("group" = "a",

"cell" = colnames(data)[i],

"cor" = cor$estimate,

"p" = cor$p.value))

}

data1 <- data1[order(data1$cor),]

data1$cell <- factor(data1$cell, levels = data1$cell)

ggplot(data1, aes(x = cell, y = cor)) +

geom_segment(aes(xend=cell,yend=0)) +

geom_hline(yintercept = 0) +

geom_point(aes(col=p, fill = p, size=abs(cor))) +

coord_flip()
