# Supplementary figures and images for: DUSP10 upregulation is a poor prognosticator and promotes cell proliferation and migration in glioma
Source: Front Oncol. 2023 Jan 11;12:1050756. doi: 10.3389/fonc.2022.1050756 (PMC9874937; doi:10.3389/fonc.2022.1050756)

The expression of DUSP10  
 $\text{Log}_2(\text{TPM}+1)$

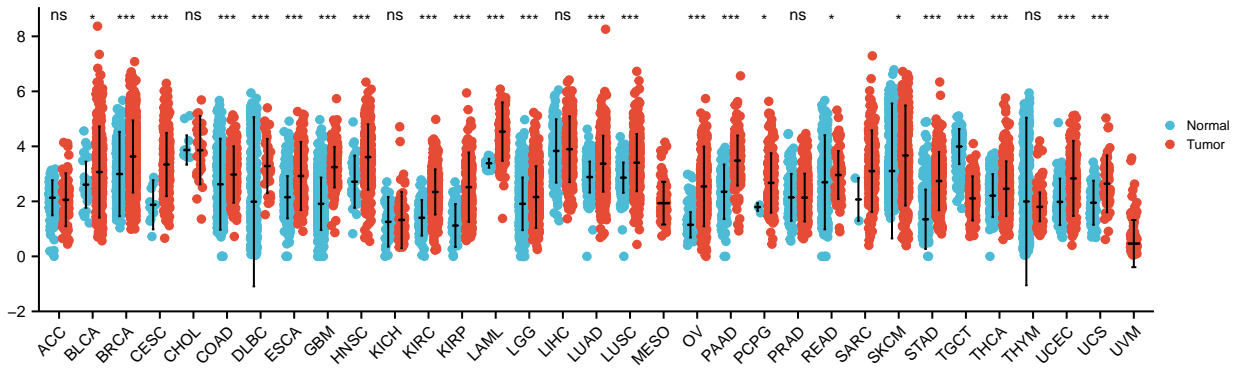

Supplement: Supplementary file 1 [file DataSheet_1.zip › DUSP10 raw data/Figure 1/1A.pdf]

The expression of DUSP10  
 $\text{Log}_2(\text{TPM}+1)$

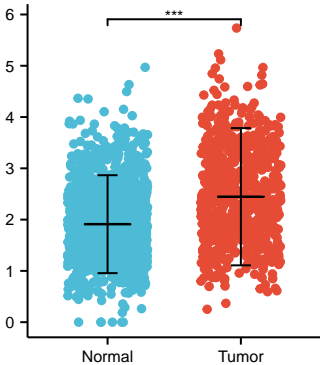

Supplement: Supplementary file 1 [file DataSheet_1.zip › DUSP10 raw data/Figure 1/1B.pdf]

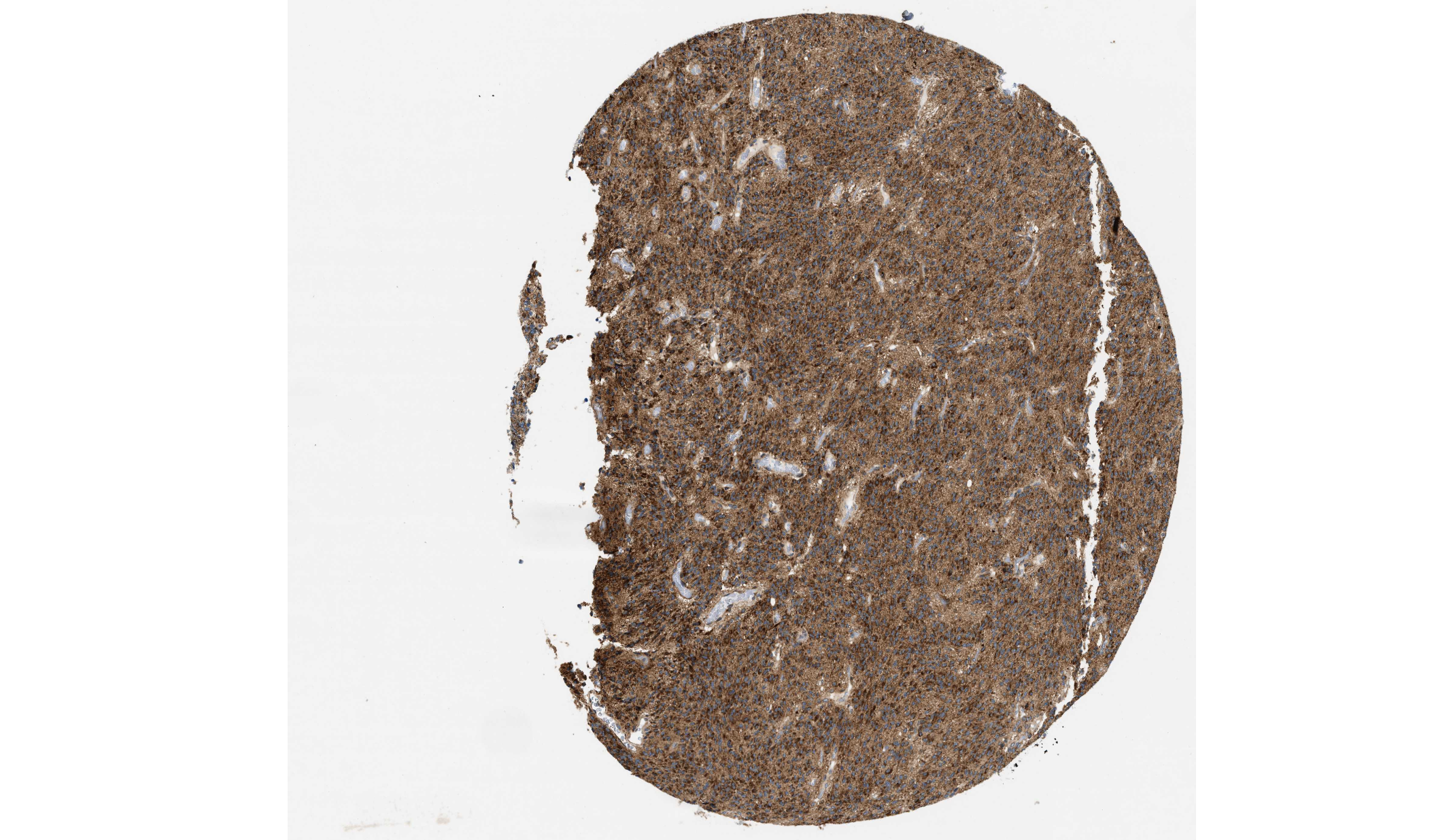

Supplement: Supplementary file 1 [file DataSheet_1.zip › DUSP10 raw data/Figure 1/1C/high.png]

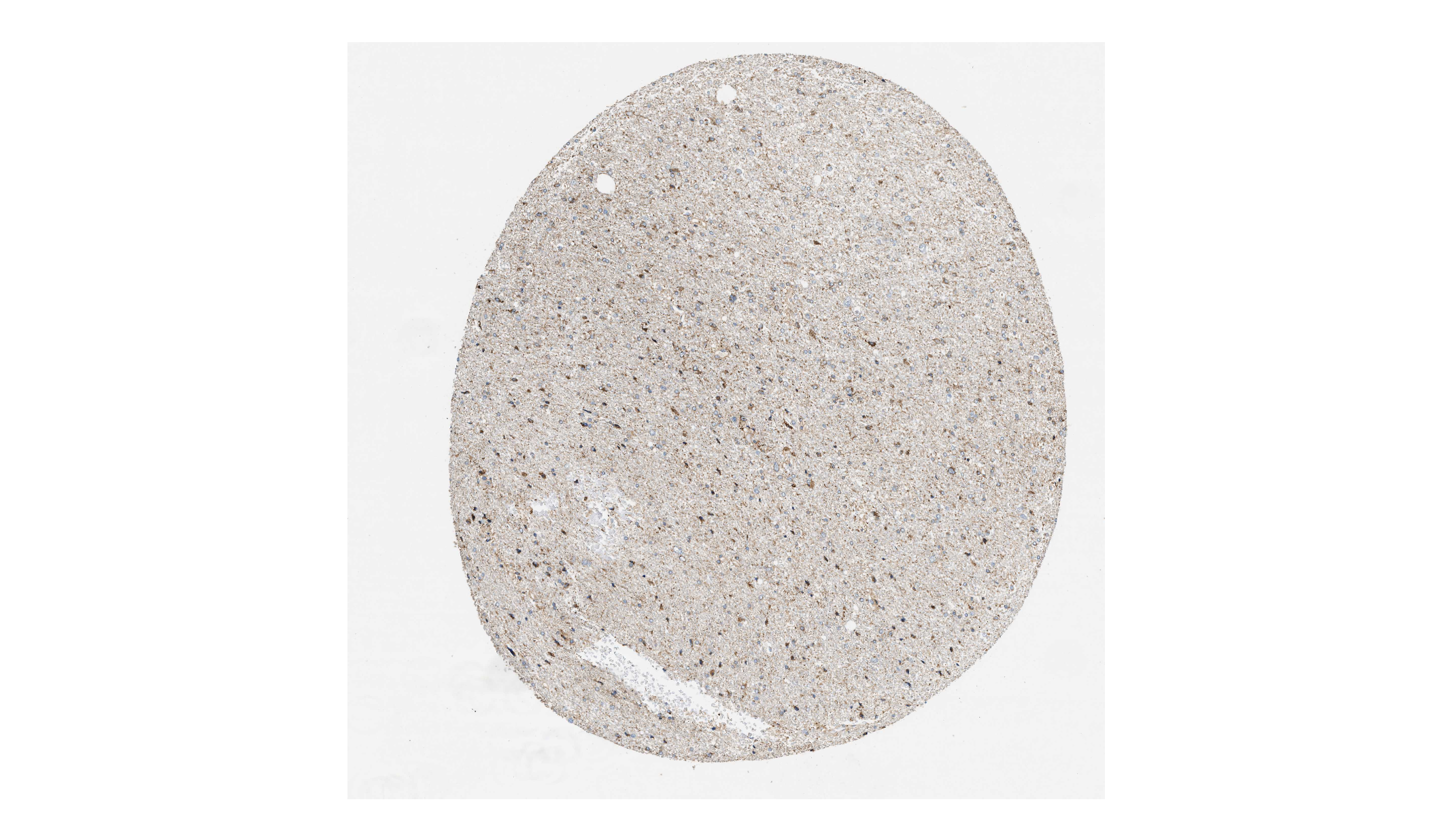

Supplement: Supplementary file 1 [file DataSheet_1.zip › DUSP10 raw data/Figure 1/1C/Low.png]

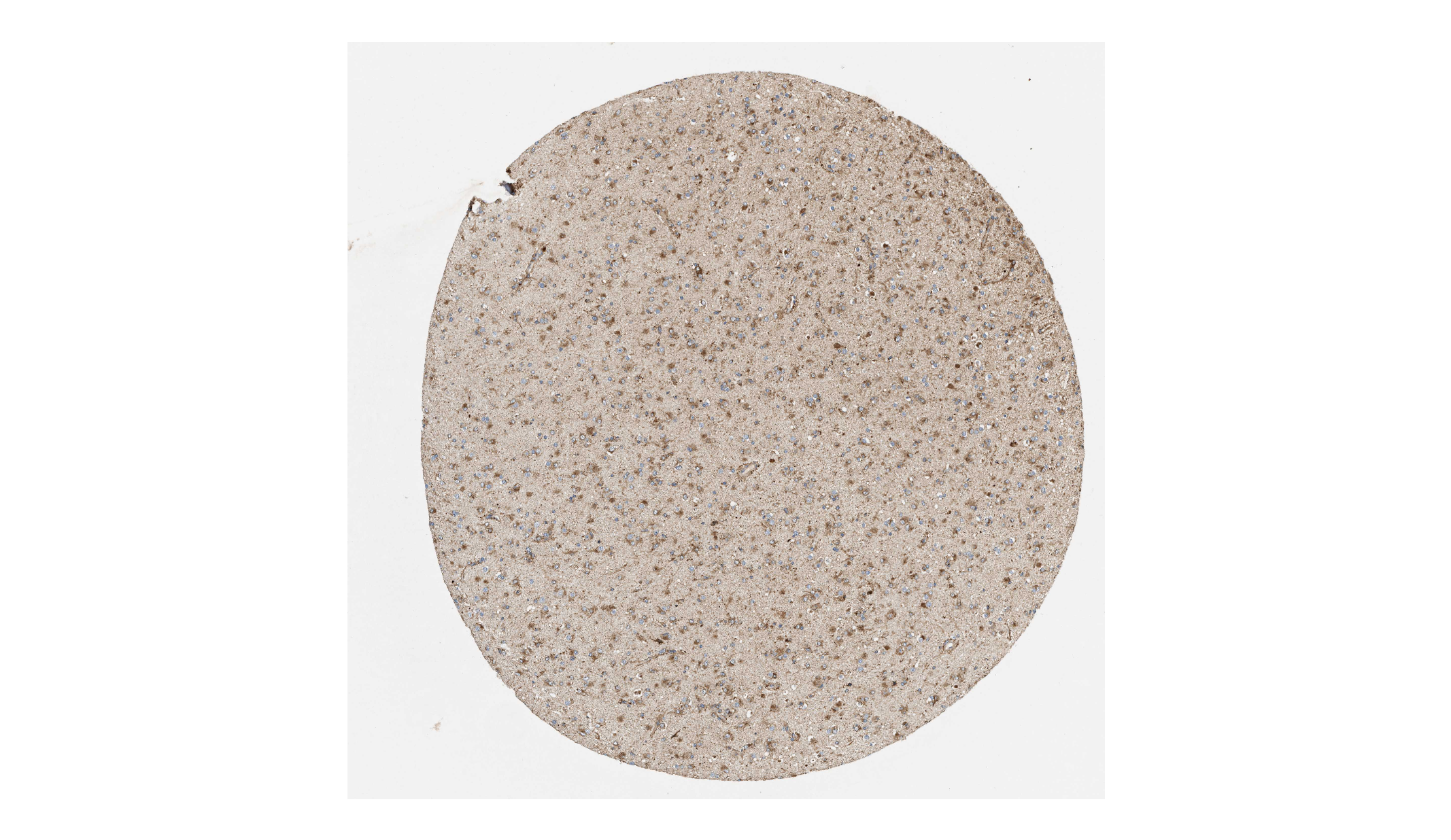

Supplement: Supplementary file 1 [file DataSheet_1.zip › DUSP10 raw data/Figure 1/1C/medi.png]

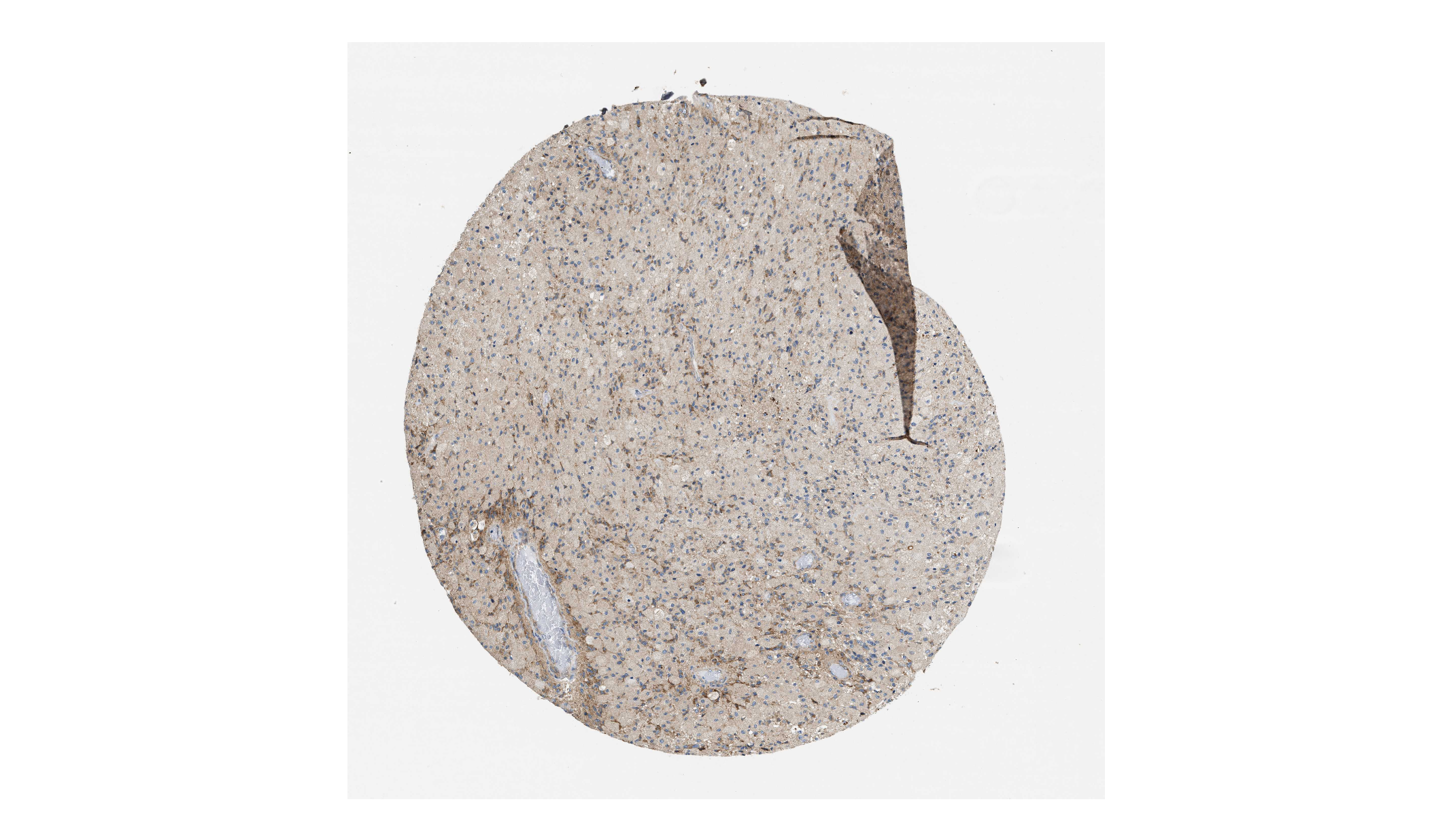

Supplement: Supplementary file 1 [file DataSheet_1.zip › DUSP10 raw data/Figure 1/1C/No.png]

The expression of DUSP10  
 $\text{Log}_2(\text{TPM}+1)$

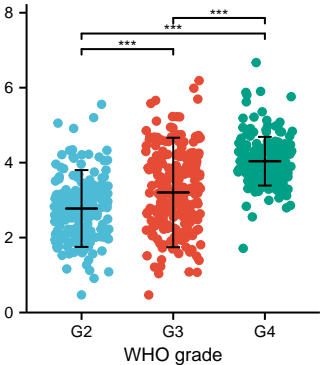

Supplement: Supplementary file 1 [file DataSheet_1.zip › DUSP10 raw data/Figure 2/2A.pdf]

The expression of DUSP10  
 $\text{Log}_2(\text{TPM}+1)$

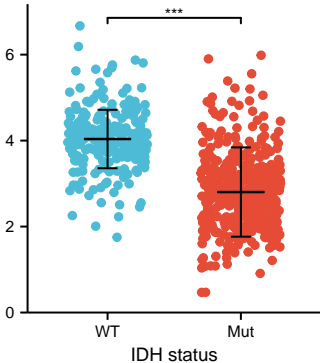

Supplement: Supplementary file 1 [file DataSheet_1.zip › DUSP10 raw data/Figure 2/2B.pdf]

The expression of DUSP10  
 $\text{Log}_2(\text{TPM}+1)$

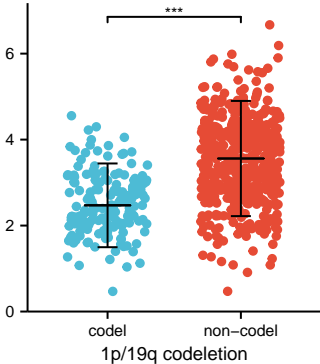

Supplement: Supplementary file 1 [file DataSheet_1.zip › DUSP10 raw data/Figure 2/2C.pdf]

The expression of DUSP10  
 $\text{Log}_2(\text{TPM}+1)$

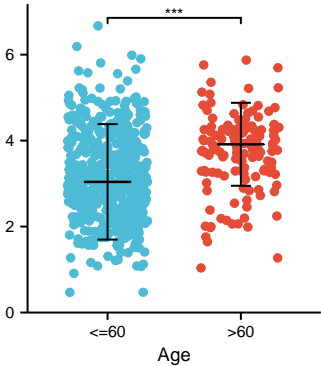

Supplement: Supplementary file 1 [file DataSheet_1.zip › DUSP10 raw data/Figure 2/2D.pdf]

The expression of DUSP10  
 $\text{Log}_2(\text{TPM}+1)$

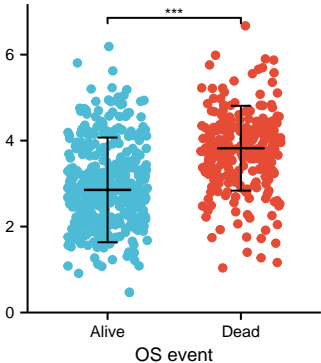

Supplement: Supplementary file 1 [file DataSheet_1.zip › DUSP10 raw data/Figure 2/2E.pdf]

The expression of DUSP10  
 $\text{Log}_2(\text{TPM}+1)$

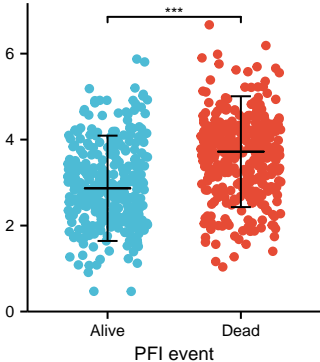

Supplement: Supplementary file 1 [file DataSheet_1.zip › DUSP10 raw data/Figure 2/2F.pdf]

The expression of DUSP10  
 $\text{Log}_2(\text{TPM}+1)$

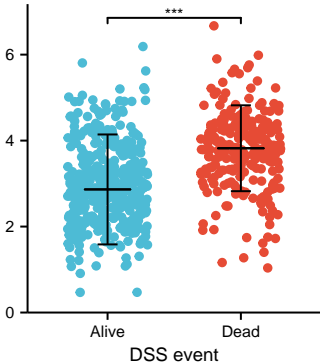

Supplement: Supplementary file 1 [file DataSheet_1.zip › DUSP10 raw data/Figure 2/2G.pdf]

The expression of DUSP10  
 $\text{Log}_2(\text{TPM}+1)$

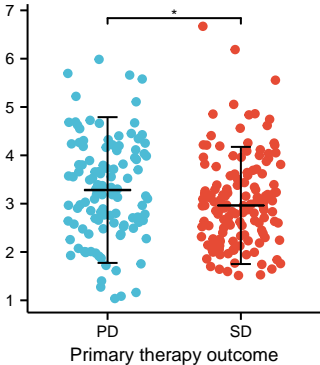

Supplement: Supplementary file 1 [file DataSheet_1.zip › DUSP10 raw data/Figure 2/2H.pdf]

The expression of DUSP10  
 $\text{Log}_2(\text{TPM}+1)$

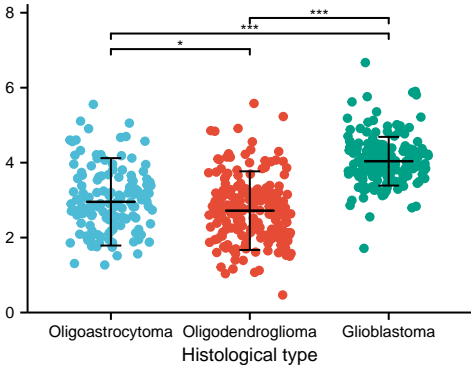

Supplement: Supplementary file 1 [file DataSheet_1.zip › DUSP10 raw data/Figure 2/2I.pdf]

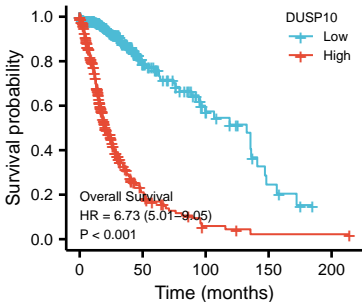

Low

|     |    |    |   |   |
|-----|----|----|---|---|
| 347 | 67 | 22 | 6 | 0 |
| 348 | 26 | 4  | 1 | 1 |

High

Supplement: Supplementary file 1 [file DataSheet_1.zip › DUSP10 raw data/Figure 3/3A.pdf]

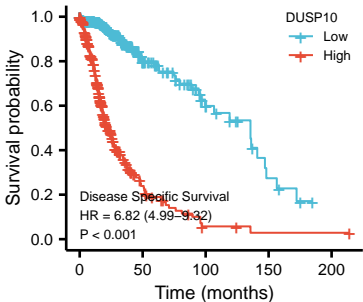

Low

|     |    |    |   |   |
|-----|----|----|---|---|
| 341 | 64 | 21 | 6 | 0 |
| 333 | 25 | 3  | 1 | 1 |

High

Supplement: Supplementary file 1 [file DataSheet_1.zip › DUSP10 raw data/Figure 3/3B.pdf]

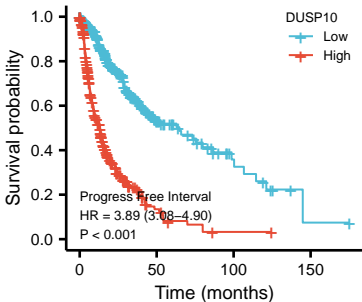

Low

|     |    |    |   |   |
|-----|----|----|---|---|
| 347 | 44 | 11 | 1 | 0 |
|-----|----|----|---|---|

High

|     |    |   |   |   |
|-----|----|---|---|---|
| 348 | 11 | 1 | 0 | 0 |
|-----|----|---|---|---|

Supplement: Supplementary file 1 [file DataSheet_1.zip › DUSP10 raw data/Figure 3/3C.pdf]

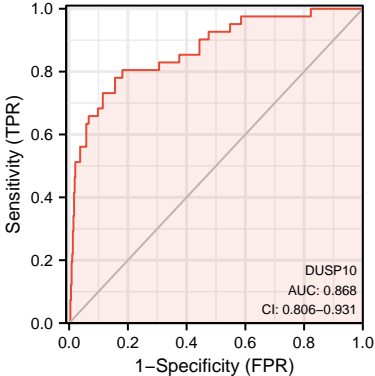

Supplement: Supplementary file 1 [file DataSheet_1.zip › DUSP10 raw data/Figure 3/3D.pdf]

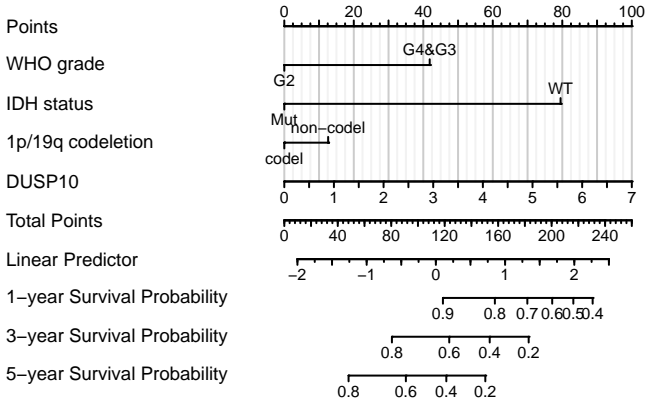

Supplement: Supplementary file 1 [file DataSheet_1.zip › DUSP10 raw data/Figure 4/4A.pdf]

Observed fraction survival probability

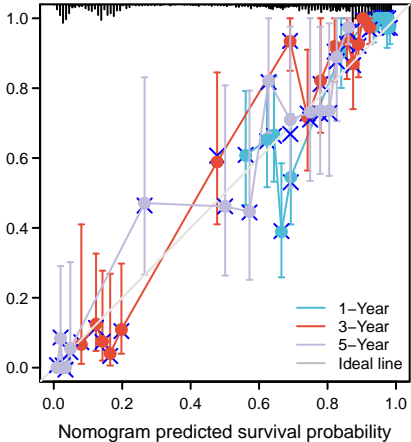

Supplement: Supplementary file 1 [file DataSheet_1.zip › DUSP10 raw data/Figure 4/4D.pdf]

Observed fraction survival probability

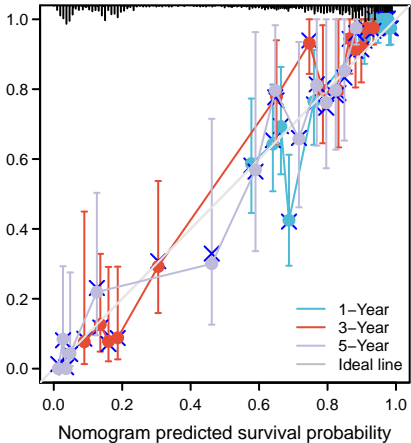

Supplement: Supplementary file 1 [file DataSheet_1.zip › DUSP10 raw data/Figure 4/4E.pdf]

Observed fraction survival probability

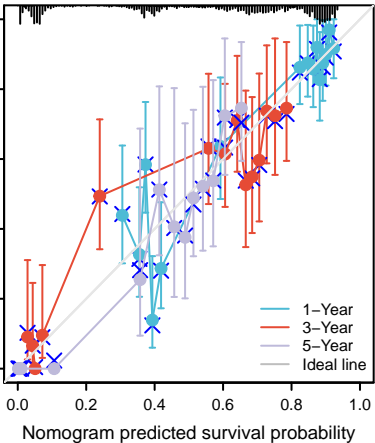

Supplement: Supplementary file 1 [file DataSheet_1.zip › DUSP10 raw data/Figure 4/4F.pdf]

# Spearman correlation between DUSP10 methylation and mRNA expression in GBM

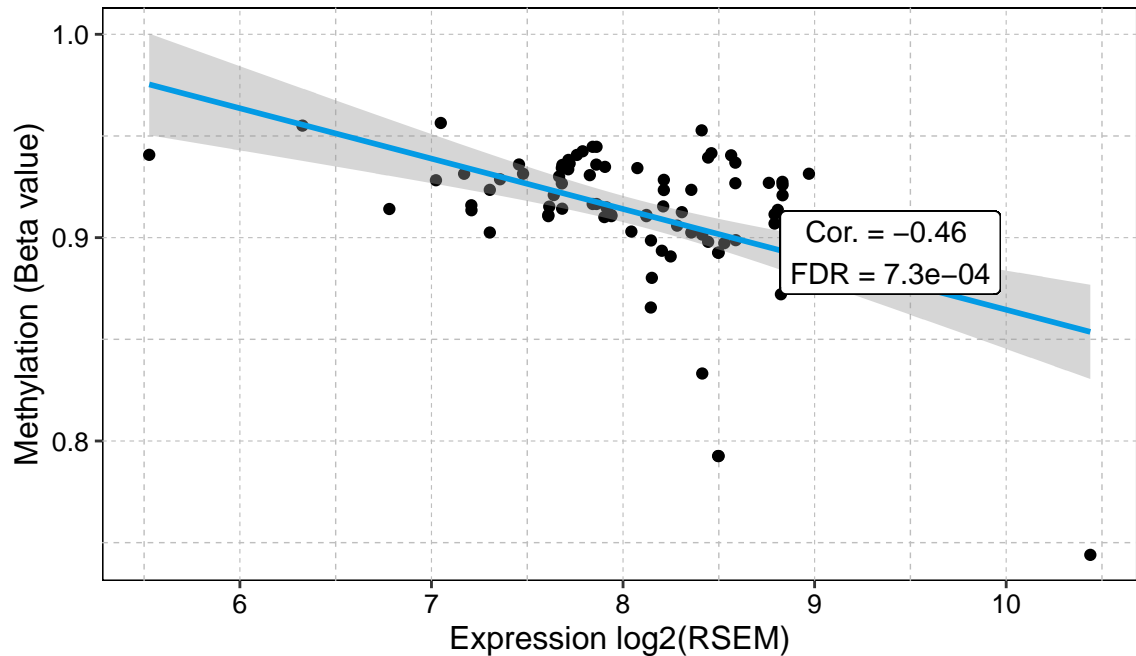

Supplement: Supplementary file 1 [file DataSheet_1.zip › DUSP10 raw data/Figure 5/5A.pdf]

# Spearman correlation between DUSP10 methylation and mRNA expression in LGG

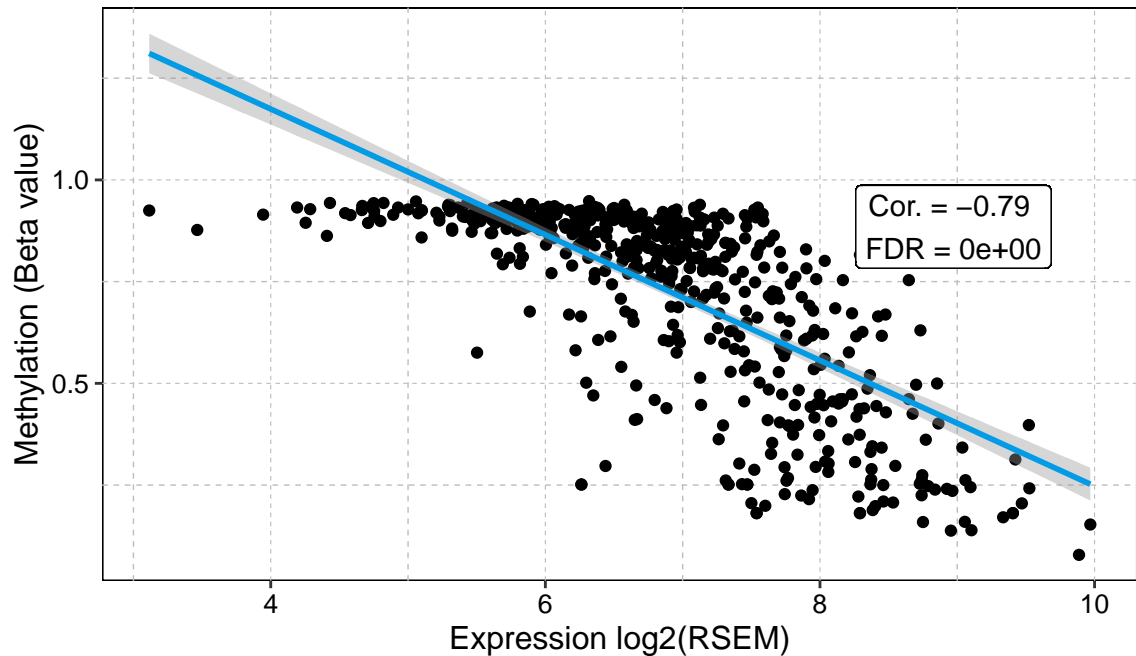

Supplement: Supplementary file 1 [file DataSheet_1.zip › DUSP10 raw data/Figure 5/5B.pdf]

# OS of DUSP10 methylation in LGG

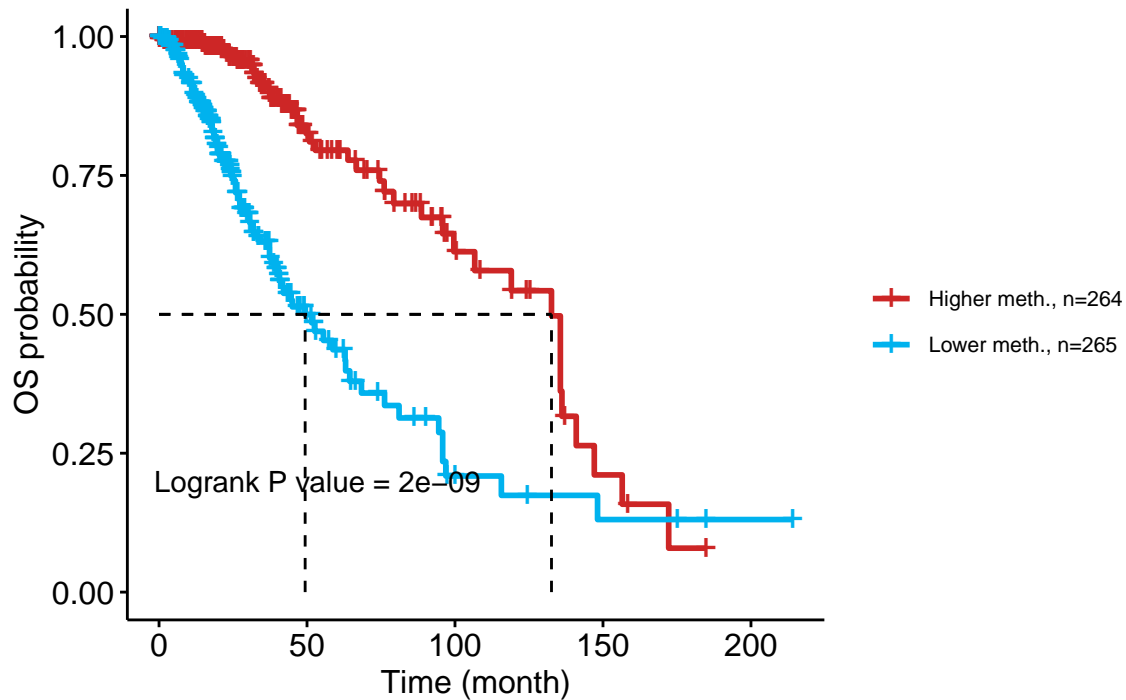

Supplement: Supplementary file 1 [file DataSheet_1.zip › DUSP10 raw data/Figure 5/5C.pdf]

# DSS of DUSP10 methylation in LGG

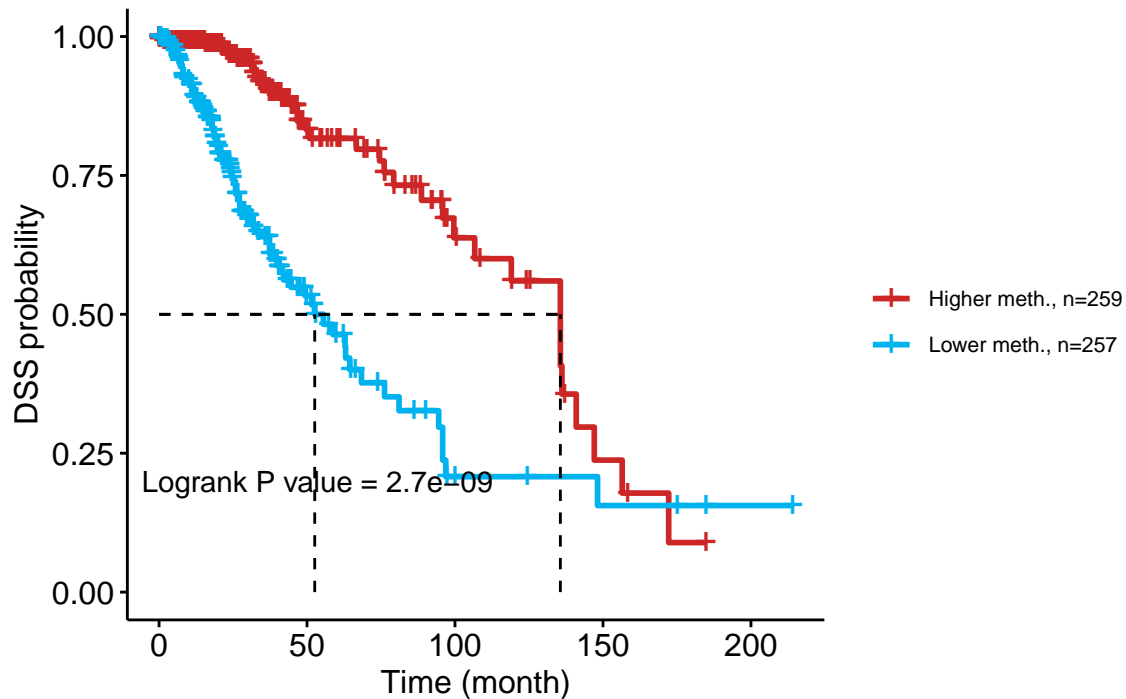

Supplement: Supplementary file 1 [file DataSheet_1.zip › DUSP10 raw data/Figure 5/5D.pdf]

# PFS of DUSP10 methylation in LGG

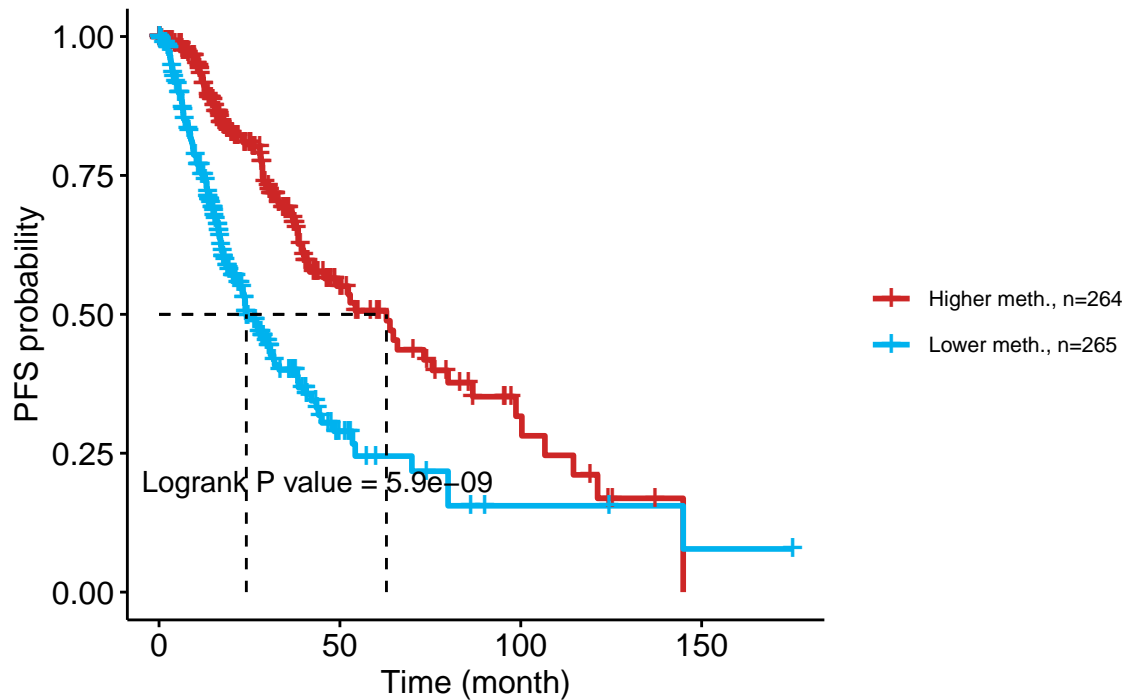

Supplement: Supplementary file 1 [file DataSheet_1.zip › DUSP10 raw data/Figure 5/5E.pdf]

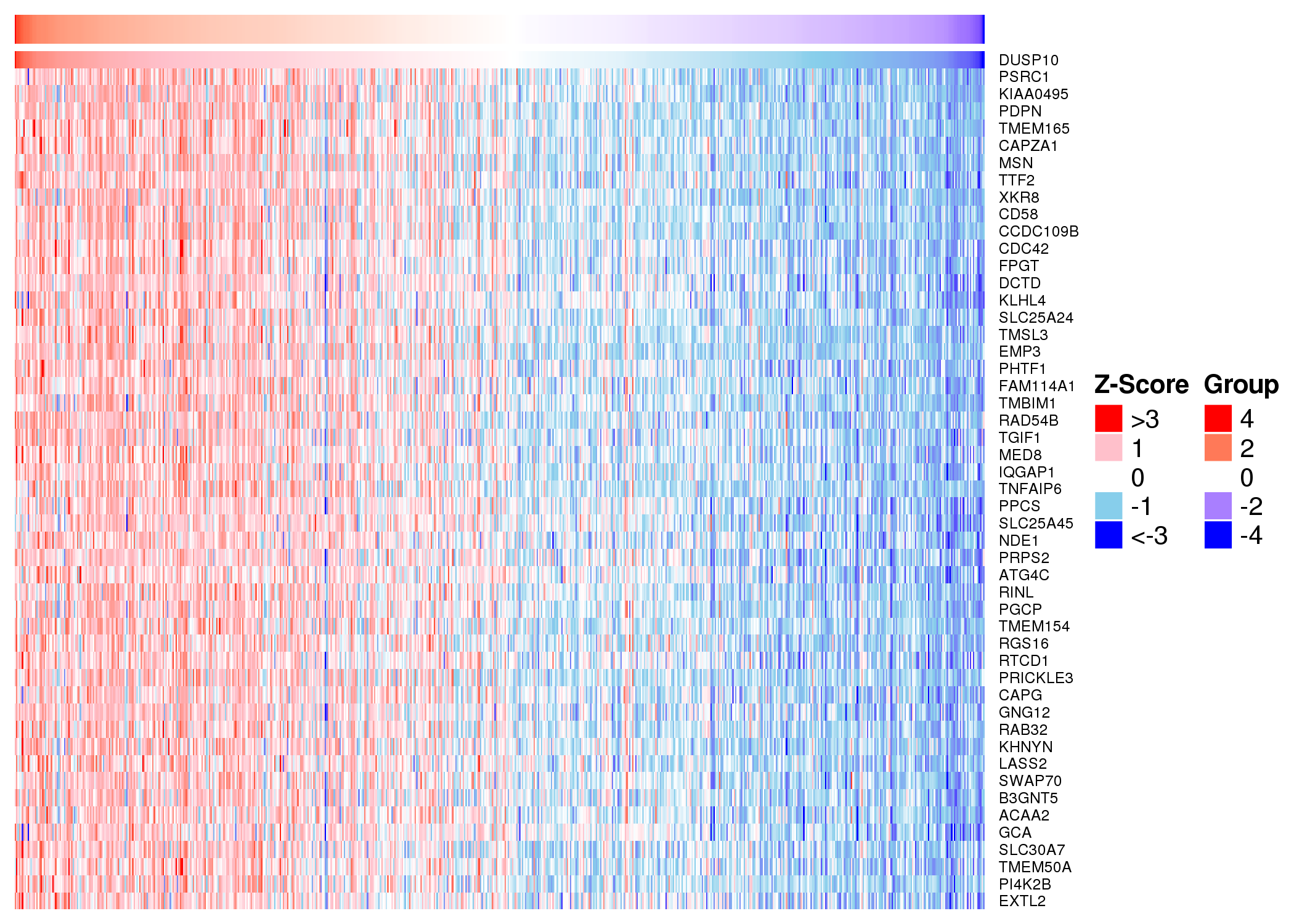

Supplement: Supplementary file 1 [file DataSheet_1.zip › DUSP10 raw data/Figure 6/6A.png]

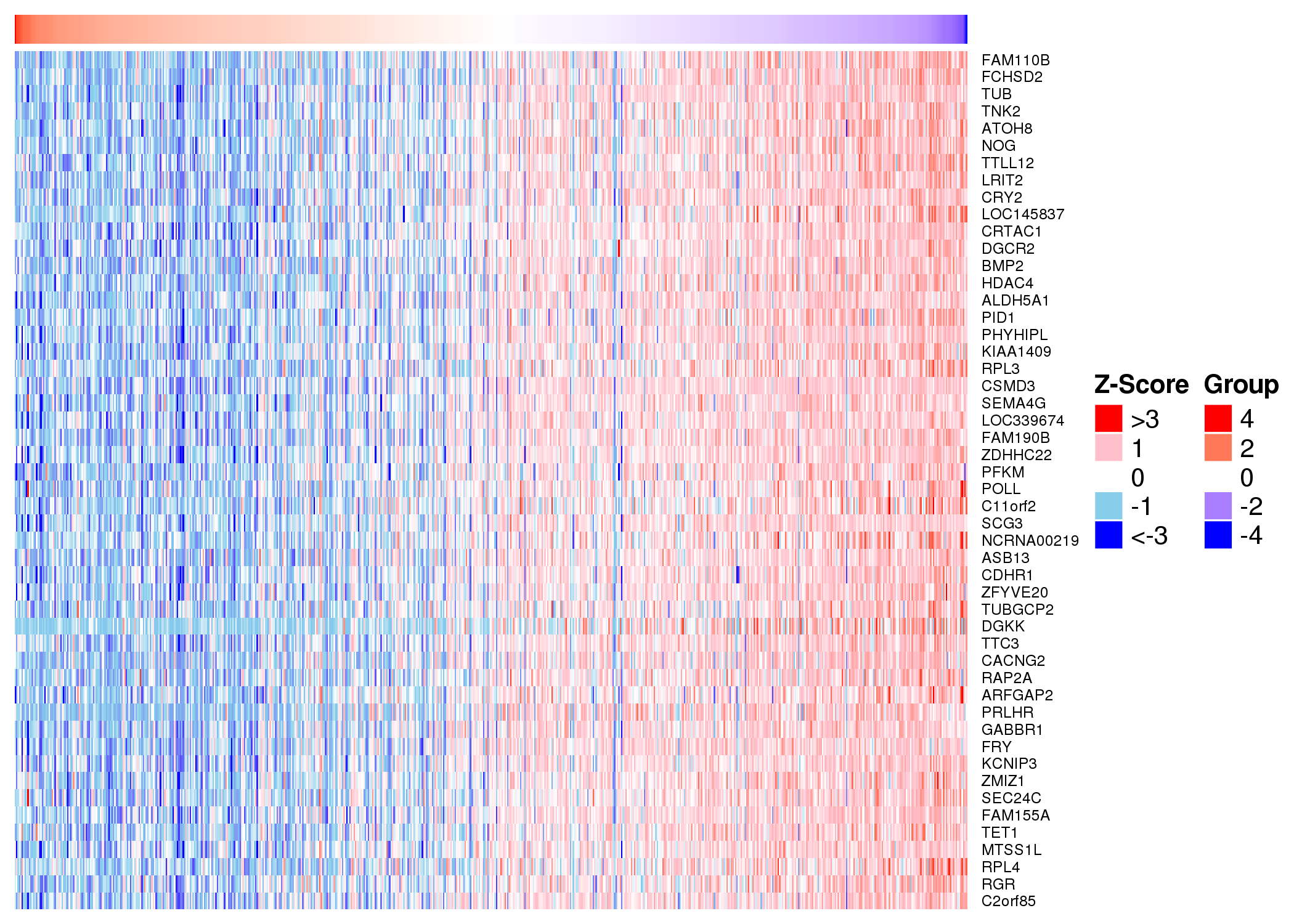

Supplement: Supplementary file 1 [file DataSheet_1.zip › DUSP10 raw data/Figure 6/6B.png]

The expression of PSRC1  
 $\text{Log}_2(\text{TPM}+1)$

8  
6  
4  
2

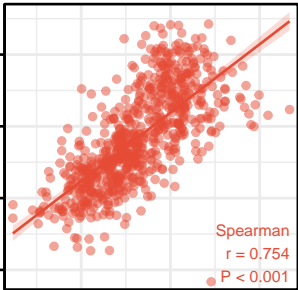

The expression of DUSP10  
 $\text{Log}_2(\text{TPM}+1)$

Supplement: Supplementary file 1 [file DataSheet_1.zip › DUSP10 raw data/Figure 6/6C/1.pdf]

The expression of PDPN  
 $\text{Log}_2(\text{TPM}+1)$

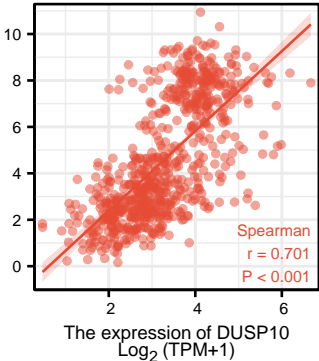

Supplement: Supplementary file 1 [file DataSheet_1.zip › DUSP10 raw data/Figure 6/6C/2.pdf]

The expression of TMEM165  
 $\text{Log}_2(\text{TPM}+1)$

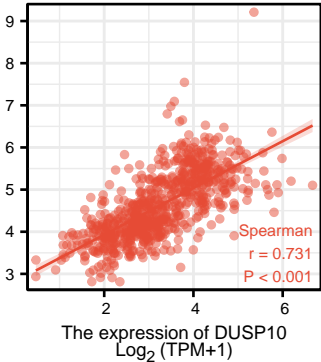

Supplement: Supplementary file 1 [file DataSheet_1.zip › DUSP10 raw data/Figure 6/6C/3.pdf]

The expression of MSN  
 $\text{Log}_2(\text{TPM}+1)$

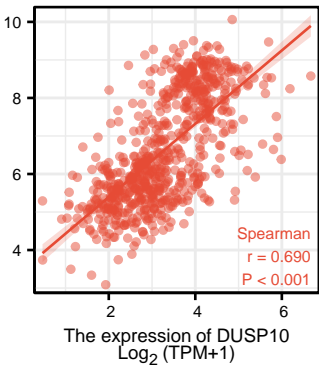

Supplement: Supplementary file 1 [file DataSheet_1.zip › DUSP10 raw data/Figure 6/6C/4.pdf]

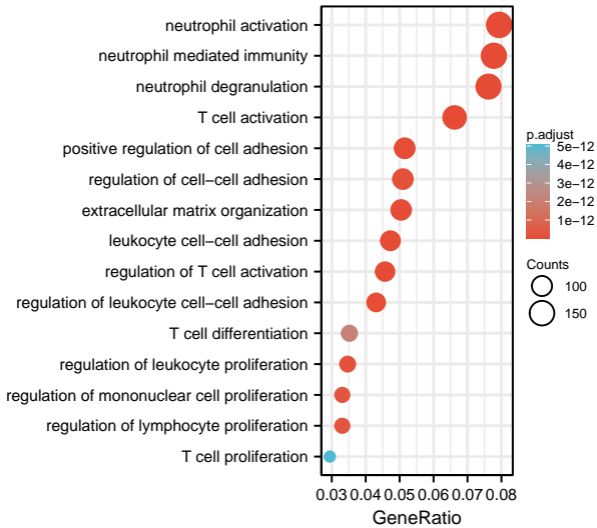

Supplement: Supplementary file 1 [file DataSheet_1.zip › DUSP10 raw data/Figure 6/6D.pdf]

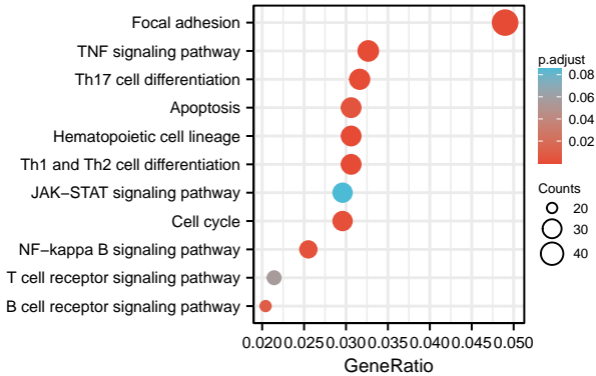

Supplement: Supplementary file 1 [file DataSheet_1.zip › DUSP10 raw data/Figure 6/6E.pdf]

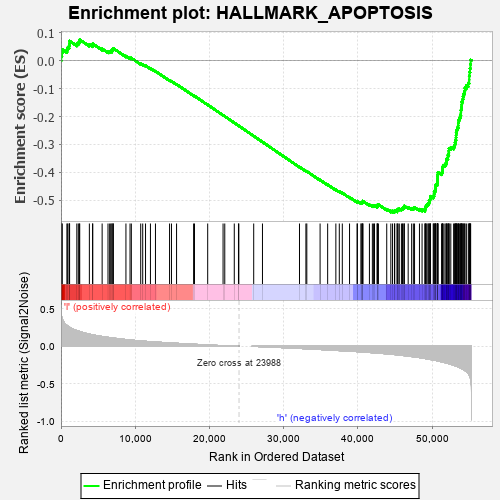

Supplement: Supplementary file 1 [file DataSheet_1.zip › DUSP10 raw data/Figure 7/enplot_HALLMARK_APOPTOSIS_36.png]

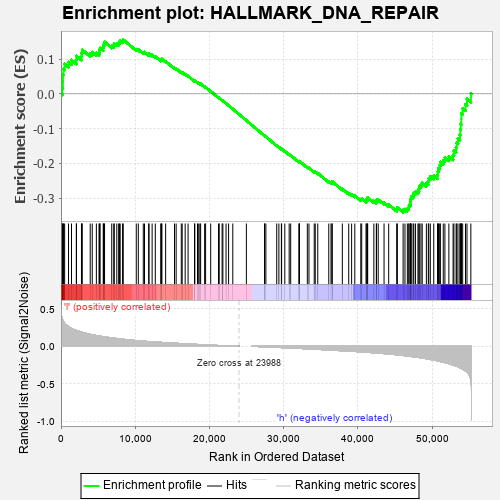

Supplement: Supplementary file 1 [file DataSheet_1.zip › DUSP10 raw data/Figure 7/enplot_HALLMARK_DNA_REPAIR_144.png]

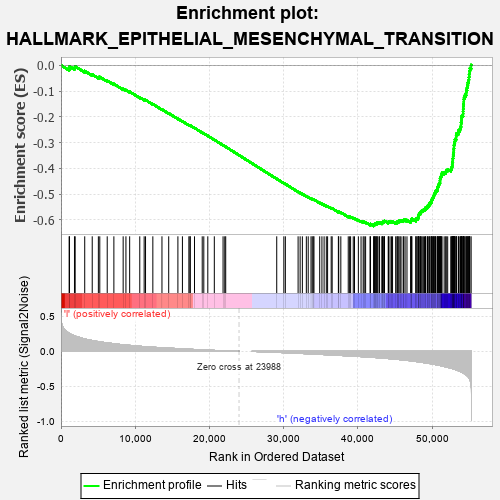

Supplement: Supplementary file 1 [file DataSheet_1.zip › DUSP10 raw data/Figure 7/enplot_HALLMARK_EPITHELIAL_MESENCHYMAL_TRANSITION_60.png]

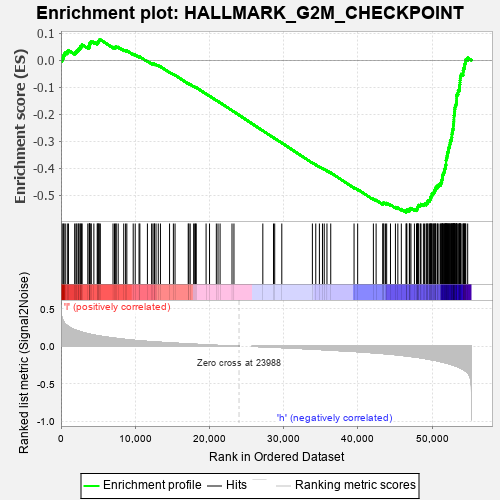

Supplement: Supplementary file 1 [file DataSheet_1.zip › DUSP10 raw data/Figure 7/enplot_HALLMARK_G2M_CHECKPOINT_117.png]

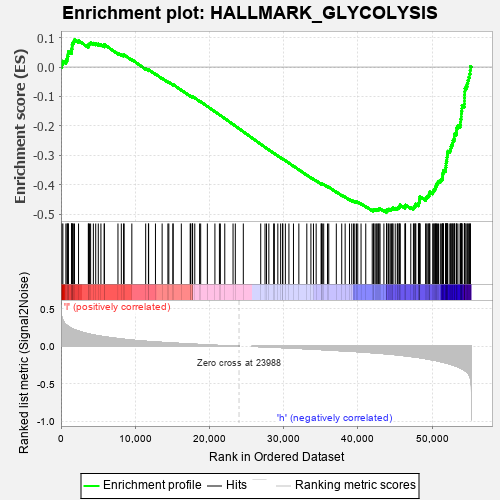

Supplement: Supplementary file 1 [file DataSheet_1.zip › DUSP10 raw data/Figure 7/enplot_HALLMARK_GLYCOLYSIS_42.png]

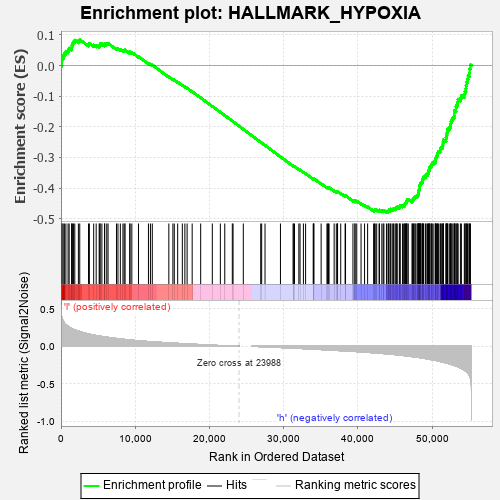

Supplement: Supplementary file 1 [file DataSheet_1.zip › DUSP10 raw data/Figure 7/enplot_HALLMARK_HYPOXIA_72.png]

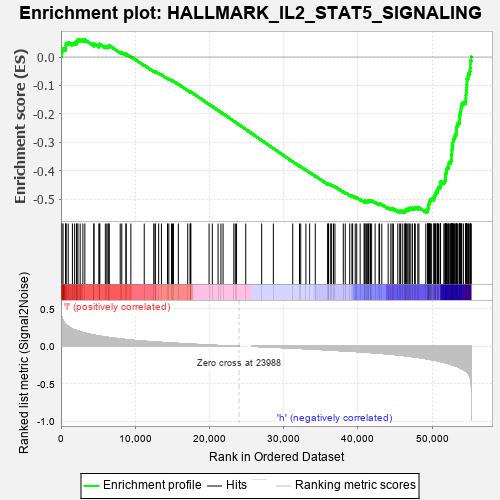

Supplement: Supplementary file 1 [file DataSheet_1.zip › DUSP10 raw data/Figure 7/enplot_HALLMARK_IL2_STAT5_SIGNALING_33.png]

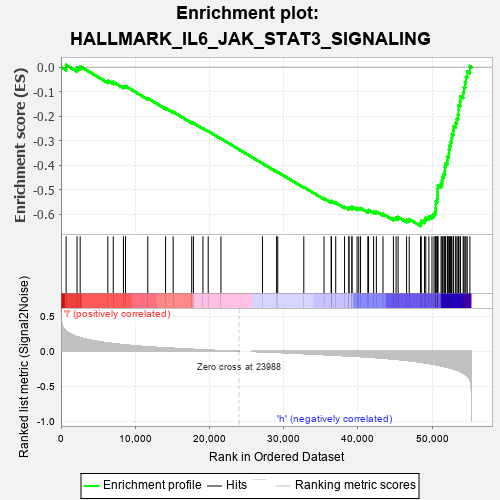

Supplement: Supplementary file 1 [file DataSheet_1.zip › DUSP10 raw data/Figure 7/enplot_HALLMARK_IL6_JAK_STAT3_SIGNALING_63.png]

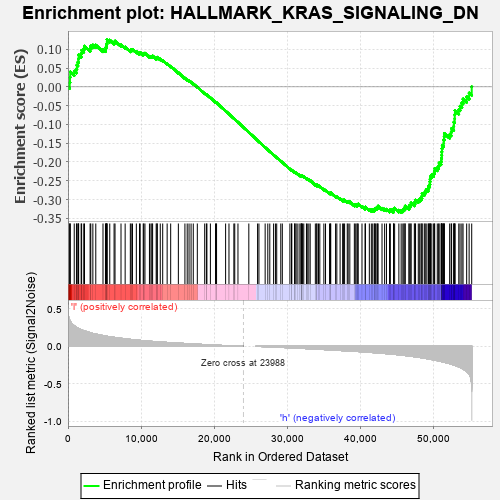

Supplement: Supplementary file 1 [file DataSheet_1.zip › DUSP10 raw data/Figure 7/enplot_HALLMARK_KRAS_SIGNALING_DN_132.png]

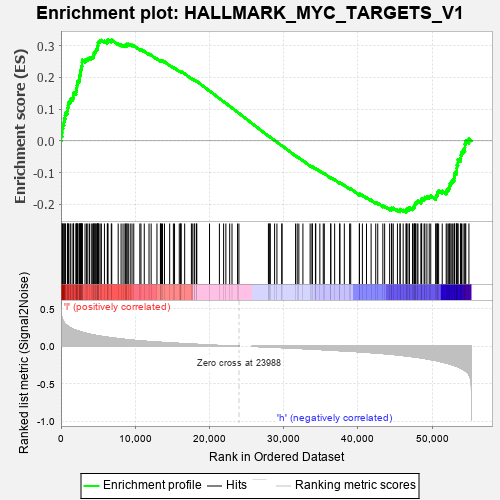

Supplement: Supplementary file 1 [file DataSheet_1.zip › DUSP10 raw data/Figure 7/enplot_HALLMARK_MYC_TARGETS_V1_12.png]

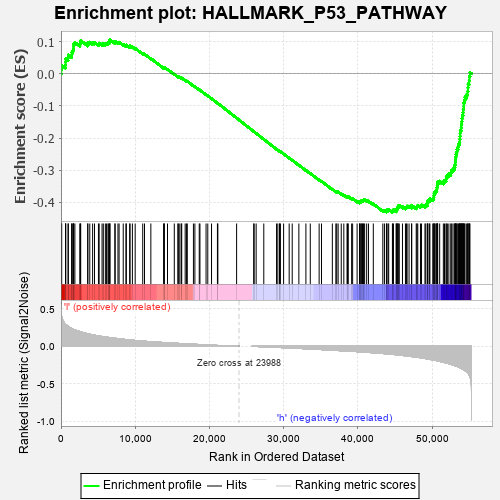

Supplement: Supplementary file 1 [file DataSheet_1.zip › DUSP10 raw data/Figure 7/enplot_HALLMARK_P53_PATHWAY_87.png]

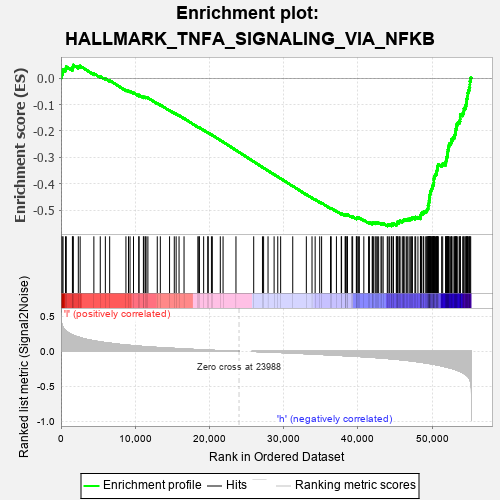

Supplement: Supplementary file 1 [file DataSheet_1.zip › DUSP10 raw data/Figure 7/enplot_HALLMARK_TNFA_SIGNALING_VIA_NFKB_93.png]

Infiltration Level

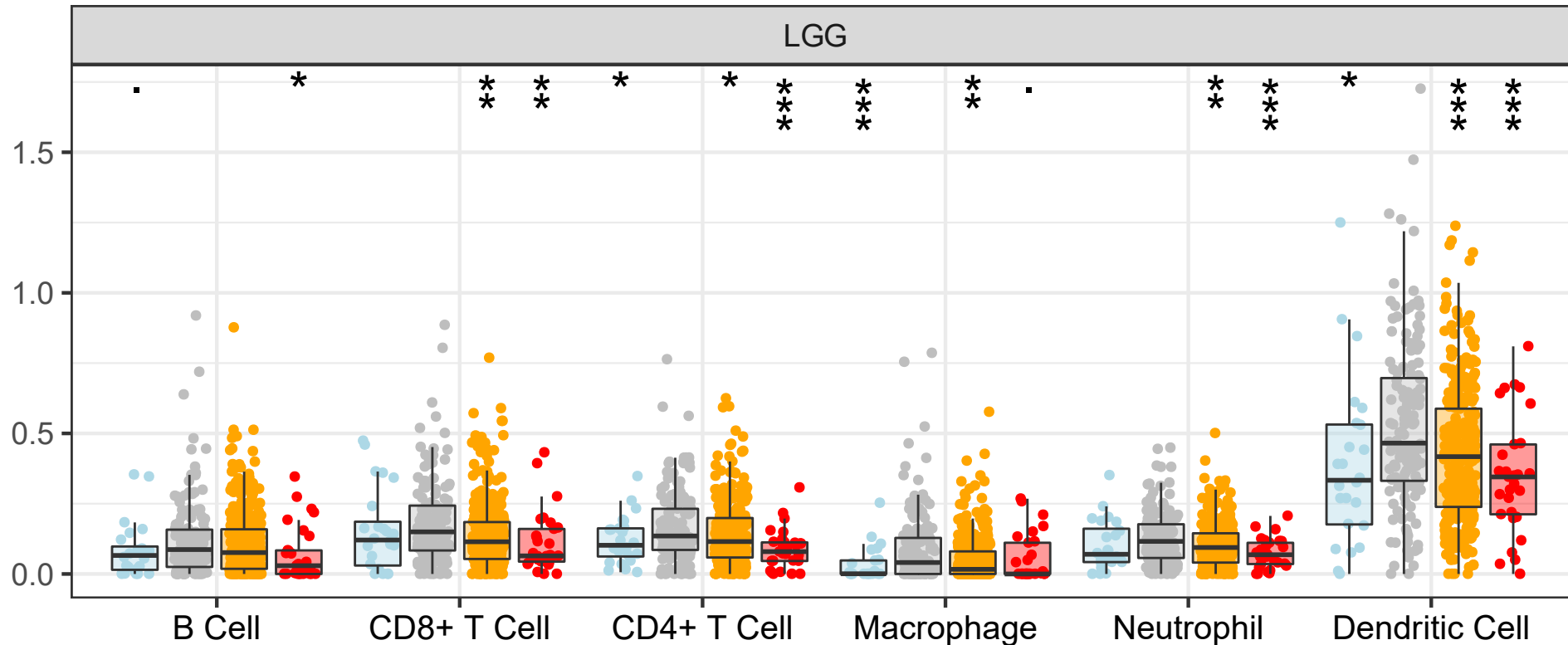

Supplement: Supplementary file 1 [file DataSheet_1.zip › DUSP10 raw data/Figure 8/8A.pdf]

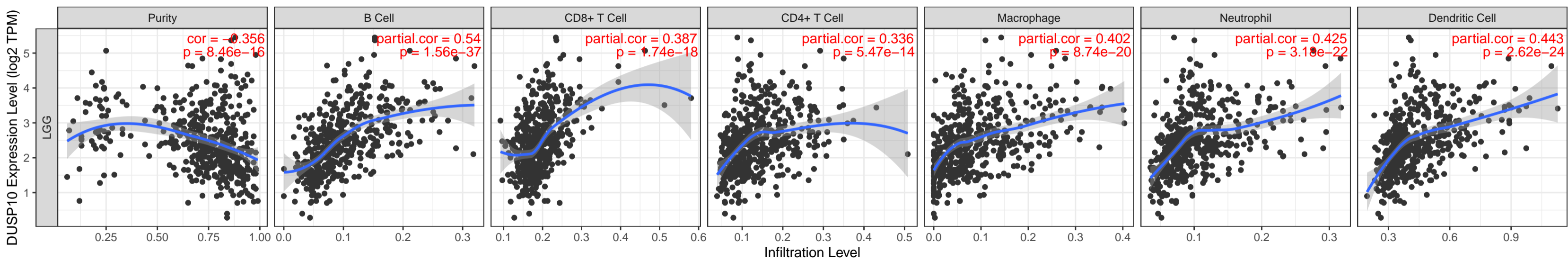

Supplement: Supplementary file 1 [file DataSheet_1.zip › DUSP10 raw data/Figure 8/8B.pdf]

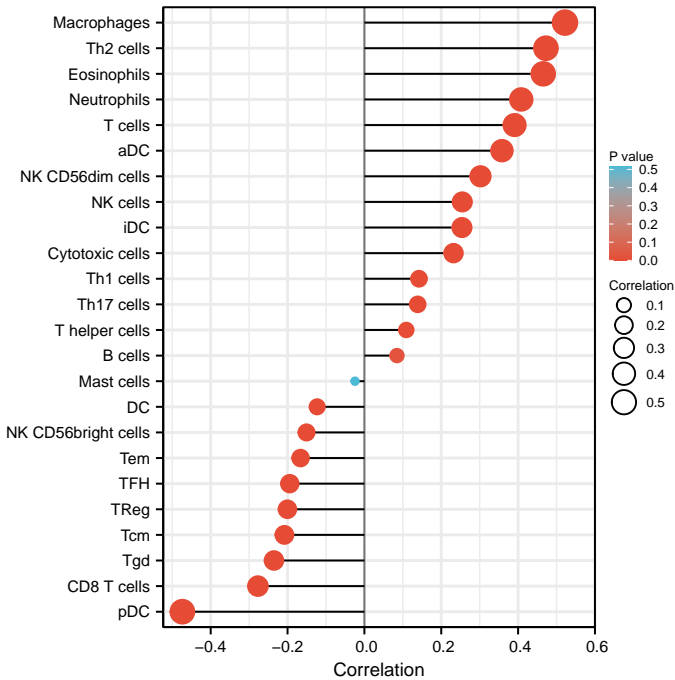

Supplement: Supplementary file 1 [file DataSheet_1.zip › DUSP10 raw data/Figure 8/8C.pdf]

DUSP10  
 $\text{Log}_2(\text{TPM}+1)$

6  
4  
2  
0

Low  
High

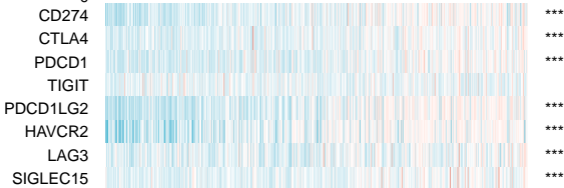

Z-score

0 4 8 12

Supplement: Supplementary file 1 [file DataSheet_1.zip › DUSP10 raw data/Figure 8/8D.pdf]

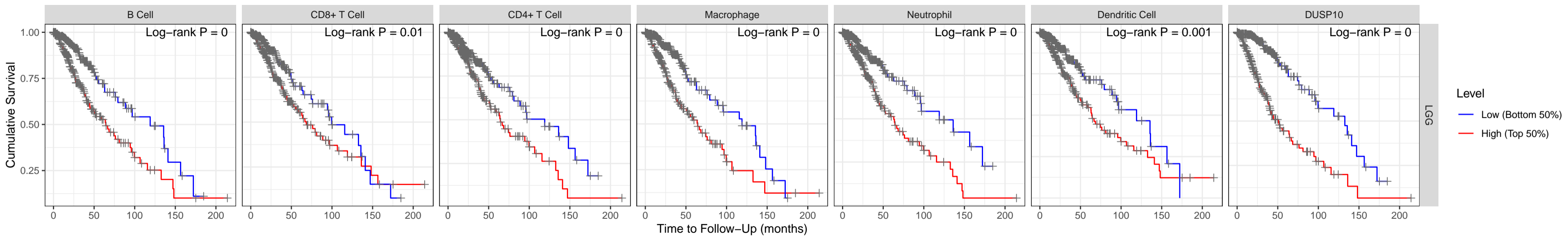

Supplement: Supplementary file 1 [file DataSheet_1.zip › DUSP10 raw data/Figure 8/8E.pdf]

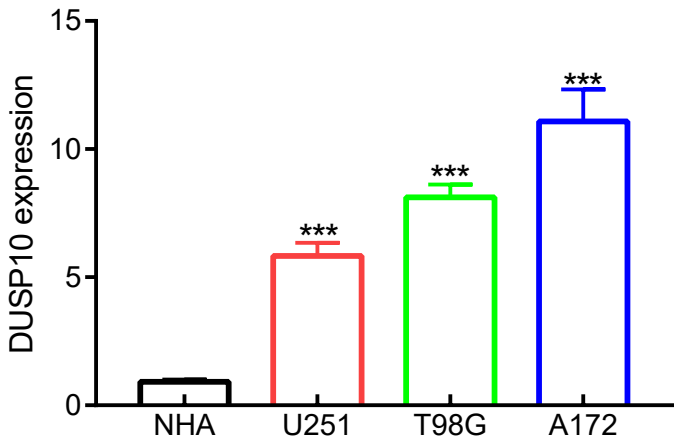

Supplement: Supplementary file 1 [file DataSheet_1.zip › DUSP10 raw data/Figure 9/9A.pdf]

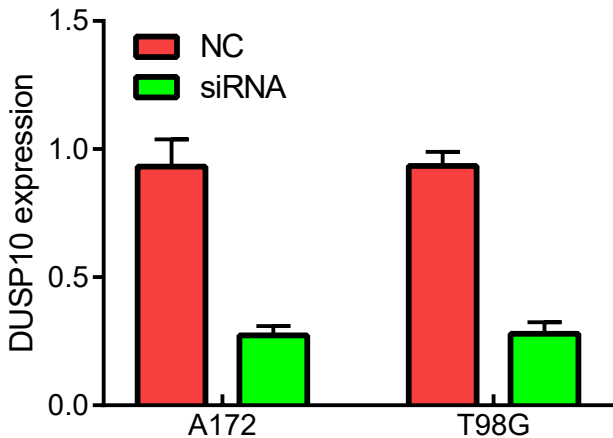

Supplement: Supplementary file 1 [file DataSheet_1.zip › DUSP10 raw data/Figure 9/9B.pdf]

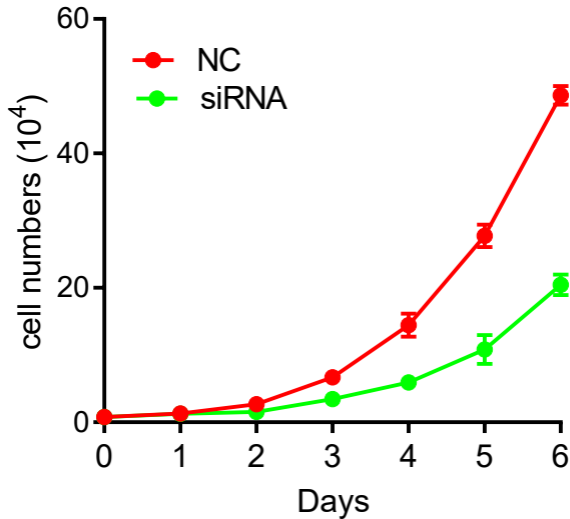

Supplement: Supplementary file 1 [file DataSheet_1.zip › DUSP10 raw data/Figure 9/9C.pdf]

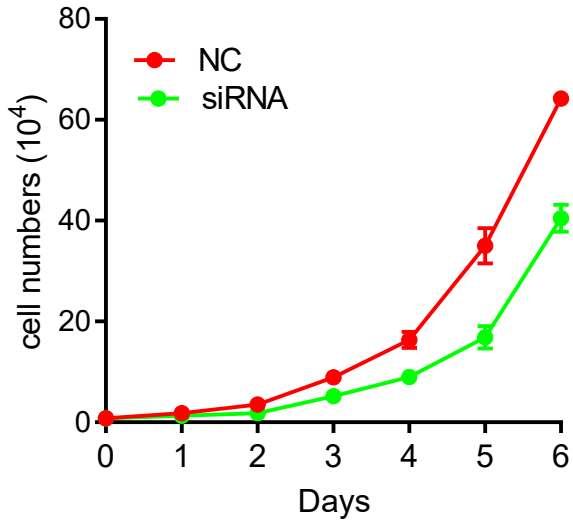

Supplement: Supplementary file 1 [file DataSheet_1.zip › DUSP10 raw data/Figure 9/9D.pdf]

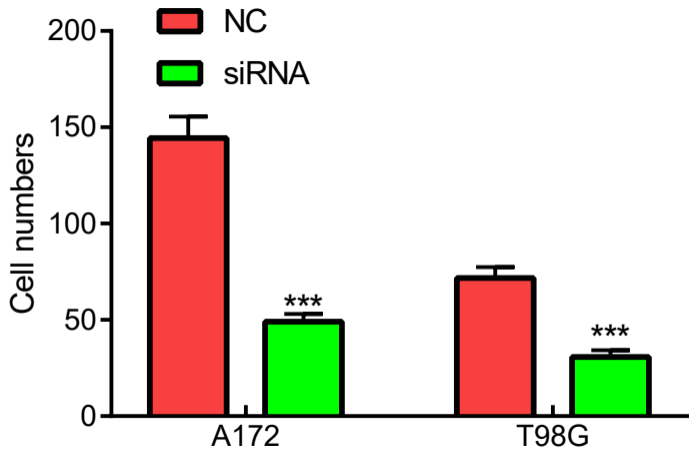

Supplement: Supplementary file 1 [file DataSheet_1.zip › DUSP10 raw data/Figure 9/9E-9F/9F.pdf]

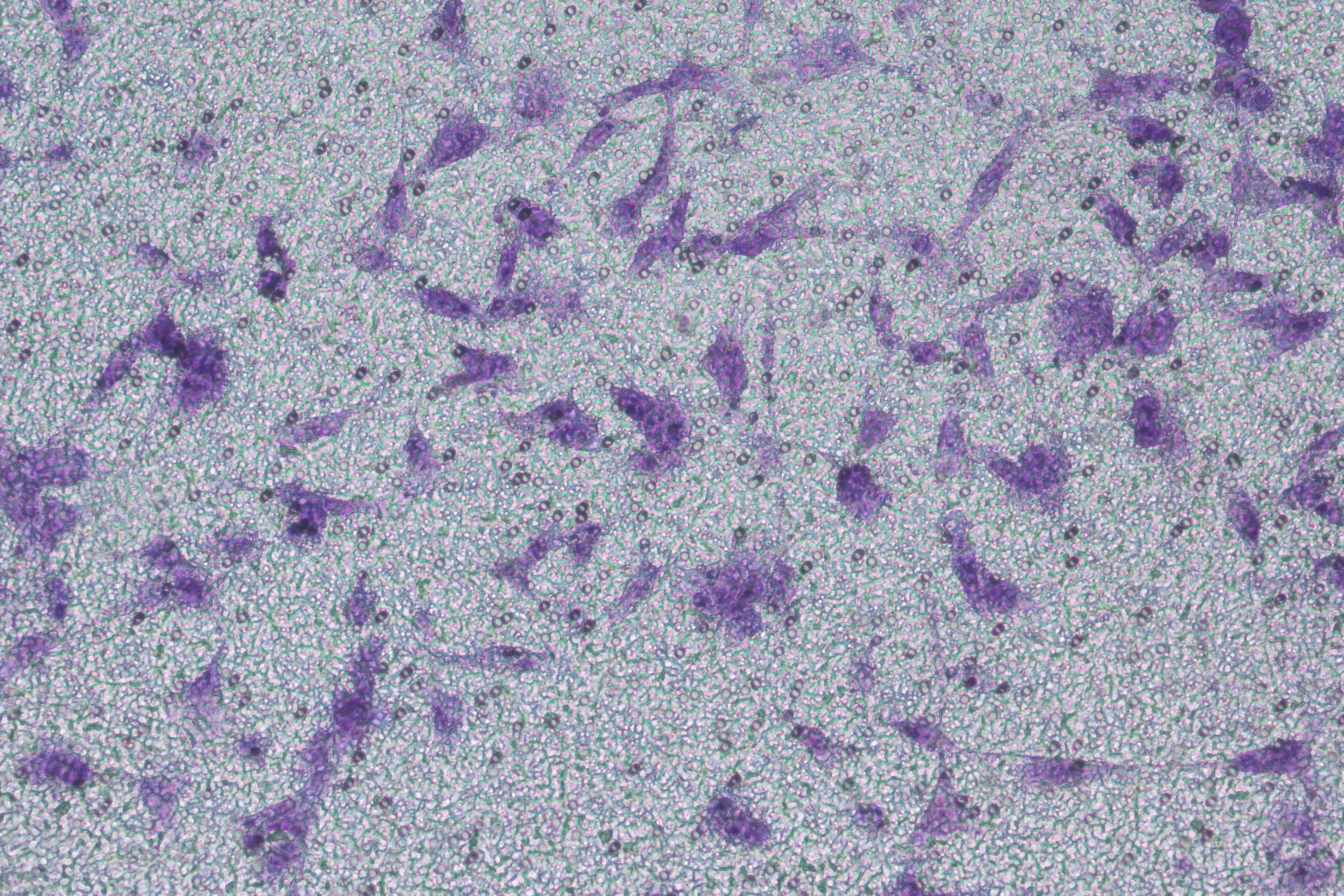

Supplement: Supplementary file 1 [file DataSheet_1.zip › DUSP10 raw data/Figure 9/9E-9F/A172/NC.jpg]

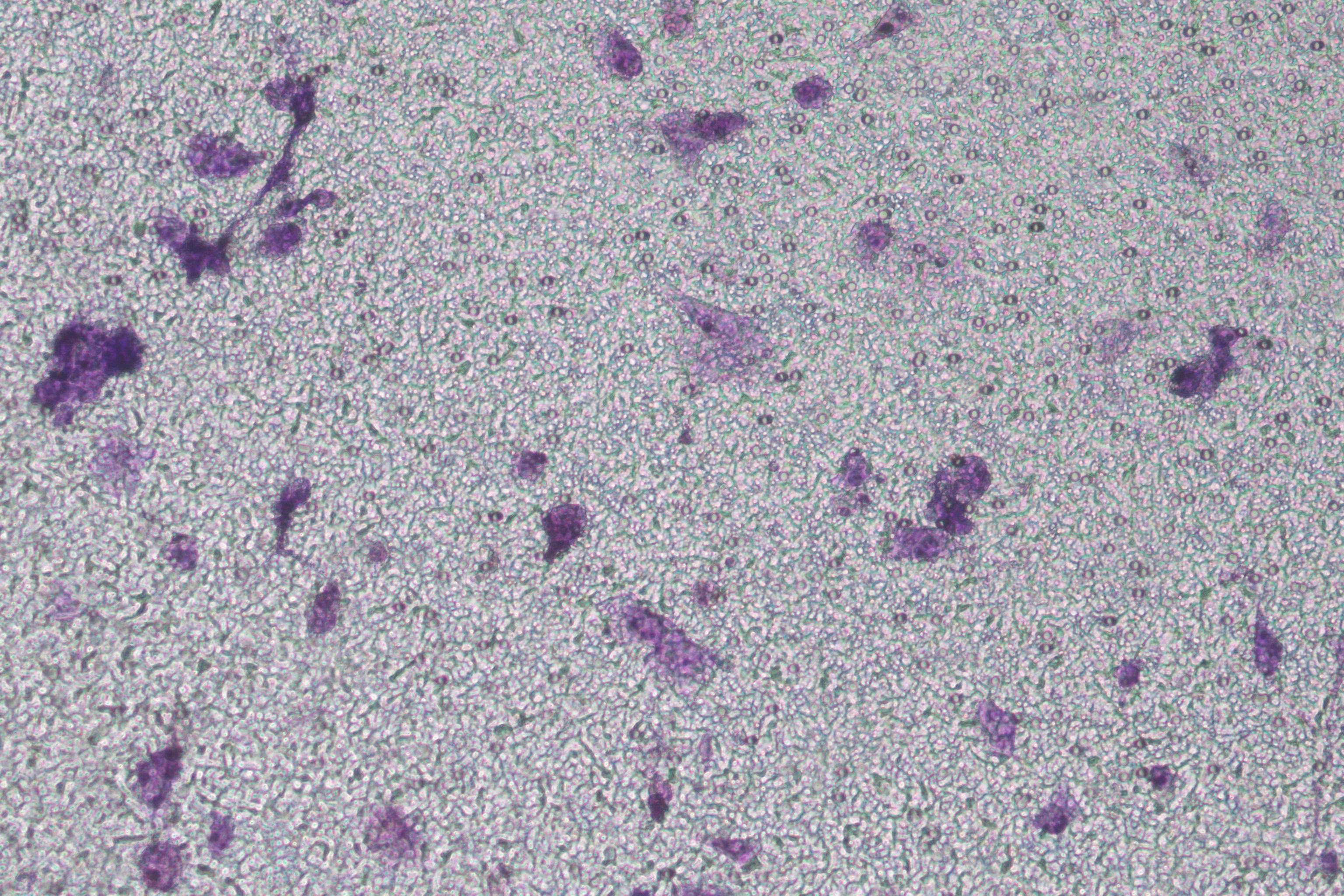

Supplement: Supplementary file 1 [file DataSheet_1.zip › DUSP10 raw data/Figure 9/9E-9F/A172/siRNA.jpg]

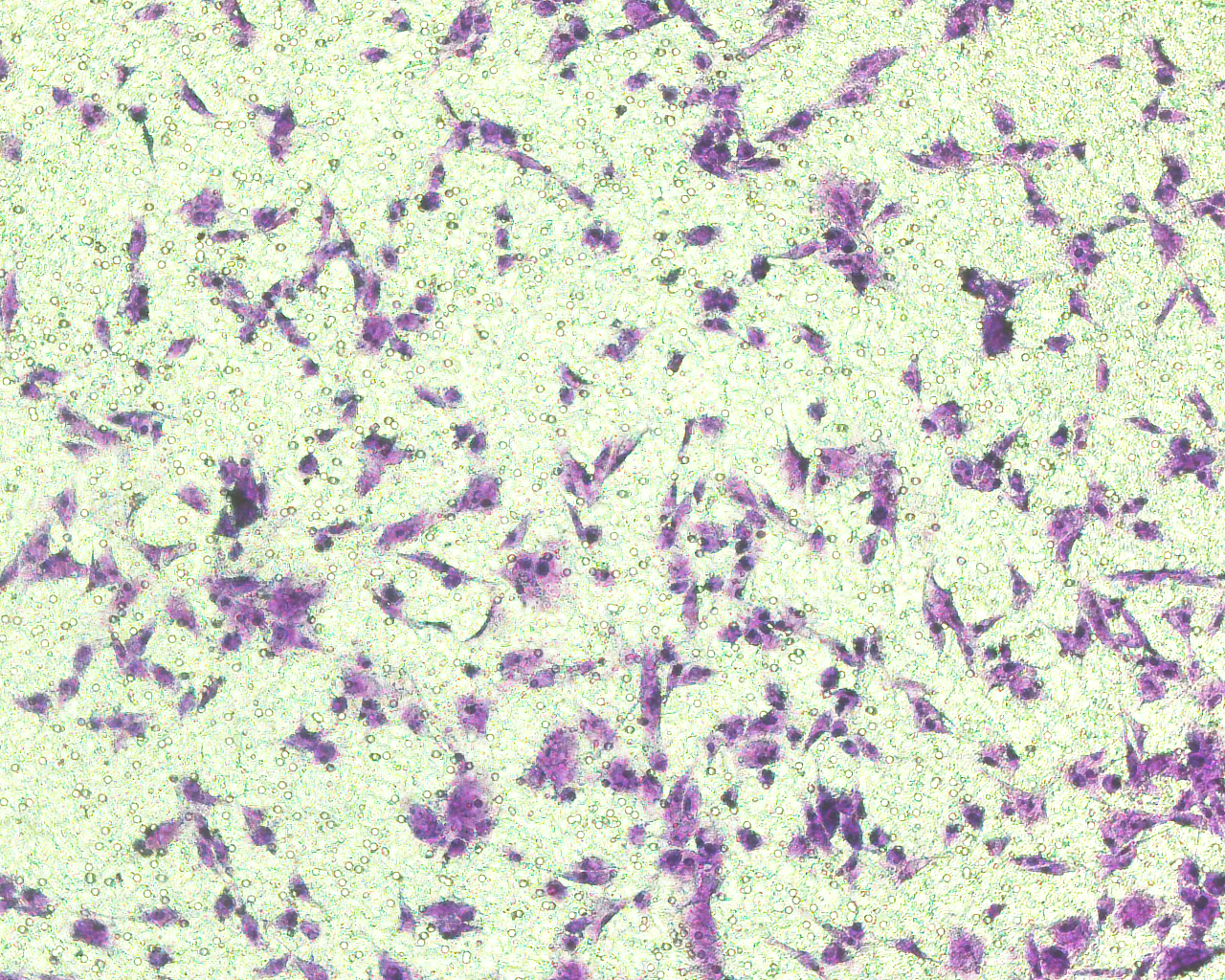

Supplement: Supplementary file 1 [file DataSheet_1.zip › DUSP10 raw data/Figure 9/9E-9F/T98G/NC.tif]

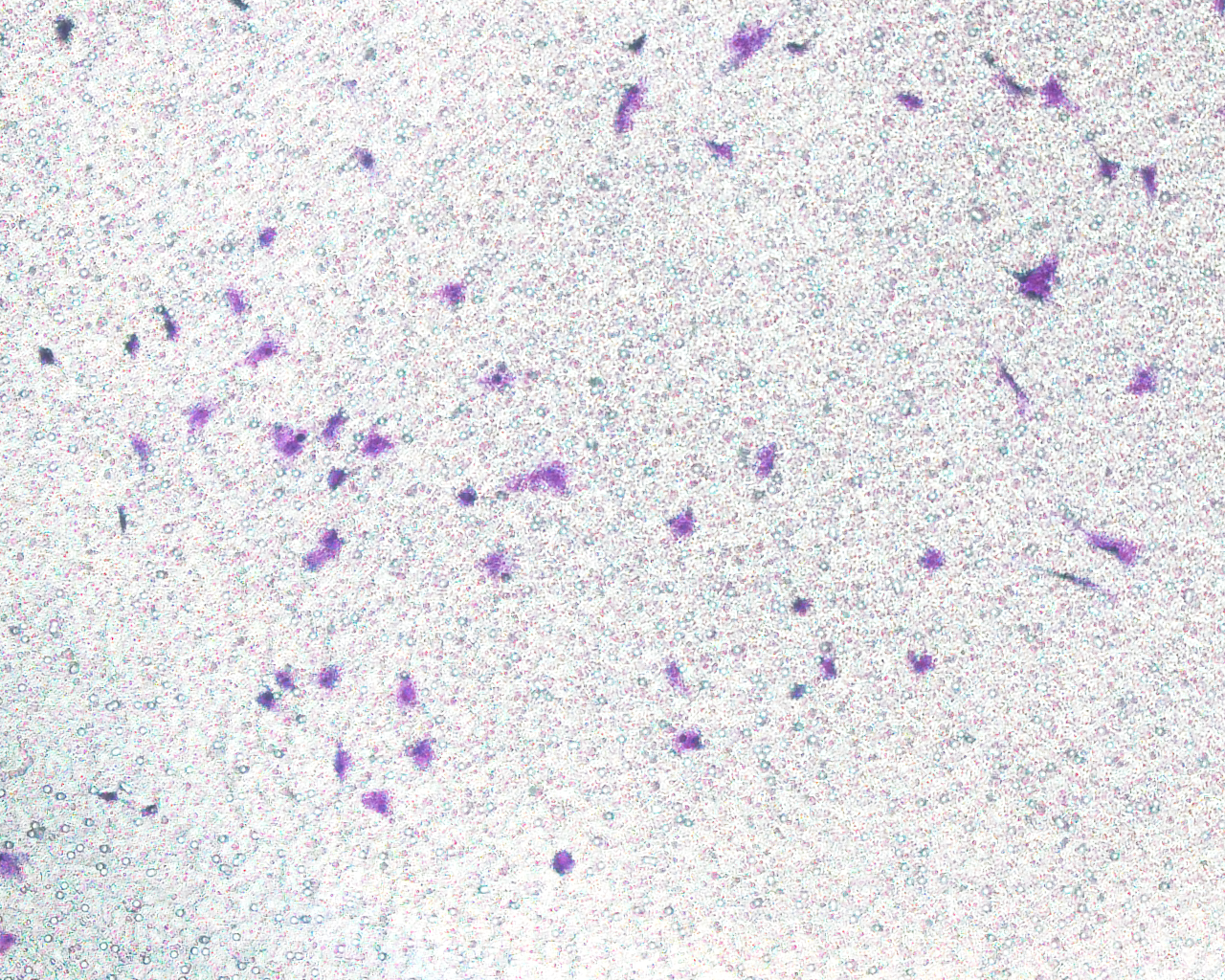

Supplement: Supplementary file 1 [file DataSheet_1.zip › DUSP10 raw data/Figure 9/9E-9F/T98G/siRNA.tif]
